# Impact analysis of flood-induced changes in geographical accessibility and coverage to healthcare in both public and private sector, 2024, Kenya

**DOI:** 10.1186/s12942-026-00461-x

**Published:** 2026-03-18

**Authors:** Bibian N. Robert, Samuel K. Muchiri, Emma W. Kahoro, Boneya H. Hindada, Helen Kiarie, Emelda A. Okiro, Peter M. Macharia

**Affiliations:** 1 Population & Health Impact Surveillance Group, Kenya Medical Research Institute- Wellcome Trust Research Programme, Nairobi, Kenya; 2 Spatial Epidemiology Lab, Université Libre de Bruxelles, Brussels, Belgium; 3 International Center for Humanitarian Affairs, Kenya Red Cross Society, Nairobi, Kenya; 4 Division of Monitoring and Evaluation, Ministry of Health, Nairobi, Kenya; 5 Centre for Tropical Medicine and Global Health, Nuffield Department of Medicine, University of Oxford, Oxford, UK; 6 Department of Public Health, Institute of Tropical Medicine, Antwerp, Belgium

**Keywords:** Geographical access, Travel time, Flooding, Healthcare system, Population affected

## Abstract

**Background:**

Climate change has caused more frequent and severe extreme weather events, threatening health system resilience worldwide. In April and May 2024, Kenya experienced unprecedented extensive floods with devastating outcomes. However, the quantitative impact of flooding on geographical access to healthcare remains unclear. This study, therefore, evaluates post-disaster accessibility to health facilities and quantifies geographical coverage losses resulting from flooding compounded by doctors’ strike in Kenya.

**Methods:**

Geospatial datasets were assembled including health facility locations (public, private not-for-profit (PNfP), and private for-profit (PfP)), road network, land use/land cover, topography, population density, and flooding extents). A pre-flood baseline and three post-flood scenarios were defined using satellite-derived flooding extents (Sentinel 1 synthetic aperture radar (SAR) and National Oceanic and Atmospheric Administration – Visible Infrared Imaging Radiometer Suite (NOAA-VIIRS) satellites) and their combined maximal extents. Travel time (TT) to the nearest health facility by type was estimated using a least-cost path algorithm, accounting for ± 20% variations in travel speed and flood extent for sensitivity analysis. Population coverage was extracted within five 30-minute TT bands for each scenario, nationally and by subnational units (county).

**Results:**

A total of 10,995 health facilities were assembled (public = 5,586; PNfP = 855; PfP = 4,554). Pre-floods, average TT to the nearest facility was 19.6 min, with public facilities at 20.7 min, PfP at 37.8 min, and PNfP at 49.2 min. Post-floods average TT increased across all sectors, longest across PNfP at 113.5 min and shortest for public facilities at 48.5 min. Pre-floods, 94.0% (52.5 million) of the population had access within 30-min and 20 out of 47 counties with an average TT of < 2 h. Under the maximal flood extents, coverage dropped to 73% (40.9 million) and only 5 counties retained < 2 h TT. County-level 30-min coverage losses ranged from 1.0% (Nairobi) to 51.0% (Narok). In several arid counties, populations facing 2 + hours TT rose to 15–31%, up from 4 to 12% pre-floods.

**Conclusion:**

Kenya’s health system is highly vulnerable to floods, causing unequal disruptions in geographical access across subnational region. Incorporating disaster preparedness into county health care planning to strengthen health system resilience nationwide is needed.

**Supplementary Information:**

The online version contains supplementary material available at 10.1186/s12942-026-00461-x.

## Introduction

Ensuring equitable healthcare access, - the ability to obtain appropriate healthcare services when needed - is crucial for improving population health outcomes [1, 2]. One of the key dimensions of healthcare access is physical accessibility, which is assessed through realised accessibility, considering actual travel time or distance, or through potential accessibility, estimating travel time accounting for barriers [2, 3]. Providing efficient access to health services is challenging for policymakers, especially during crises like natural disasters, when demand increases and the environment becomes complex. In such times, increased demand and a compromised environment make physical proximity to services a key determinant of survival and effective response, where assessments based on hazards, exposure, and vulnerabilities guide targeted measures to boost population resilience and protect critical services and infrastructure [4–6].

The increasing frequency and severity of extreme weather events, a direct consequence of climate change, pose significant threats to the resilience of health systems worldwide [7]. This threat is magnified in Africa by underlying structural vulnerabilities, including strained health infrastructure and gaps in adaptive capacity. Climate change is exacerbating vulnerabilities through disasters such as floods, droughts, and heat waves [8], while diminishing the capacity to recover from these events [9]. Floods stand out as the most prevalent natural disasters in sub-Saharan Africa (SSA) accounting for 60% of the reported natural disaster between 1970 and 2019 [10]. By 2023, floods were responsible for nearly 75% of the 6.3 million people displaced due to disasters in Africa [11]. In 2024 alone, extreme rainfall affected 27 countries, resulting in widespread flooding that impacted over 11 million people, caused about 2,500 fatalities, and displaced 4 million individuals [12]. These events have been reported to disrupt healthcare services and damage critical infrastructure [13] hindering care delivery and underscores the urgent need to bolster health system resilience [14] to anticipate, prepare for, and respond to climate-related disruptions.

In April–May 2024, Kenya faced one of its most devastating floods in decades, with over 300 fatalities and hundreds of thousands displaced [15, 16]. The risk of cholera outbreaks and an increase in malaria cases was heightened due to stagnant waters, limited access to safe water and sanitation, and the overcrowding of under-resourced, haphazardly delineated temporary shelters [17]. The floods caused substantial damage to numerous health facilities, schools and critical infrastructure [15, 18, 19]. Various sources have also estimated considerable general losses to infrastructure and personal property [16, 20, 21], but the exact quantitative impact of the flooding on geographical accessibility to healthcare remains unclear.

Modelling geographic accessibility and population coverage based on travel time to health facilities offers insights for disaster preparedness and response. Recent maps of access in Kenya highlight marginalised groups [2], and advances in remote sensing, geospatial technology, and post-disaster data collection allow accurate impact assessments, considering infrastructure damage, loss of road access, and movement barriers [5, 6, 17]. However, disaster management frameworks often lack guidance on integrating spatial barriers into accessibility assessments. Despite guidelines for post-disaster needs [22], some static measures don’t address dynamic accessibility changes caused by barriers like floods. Geographical models can identify gaps, support decision-making, and improve disaster response efficiency [4].

Here, geospatial modelling approaches were used [4] to assess post-disaster accessibility to health facilities and analyse accessibility coverage losses resulting from extreme flooding in Kenya. The approach enables the precise quantification of disaster impacts on geographical healthcare accessibility, thereby providing invaluable insights for post-disaster interventions and resilience-building efforts. Specifically, travel time before (business-as-usual -BAU) and after flooding were modelled and the proportion of the population that lost access to healthcare were quantified. Flood zones based on two independent satellites were defined and facilities were disaggregated by four ownership levels and sensitivity analyses were done based on travel speeds.

## Methods

### Kenya-Country context

**Fig. 1:**
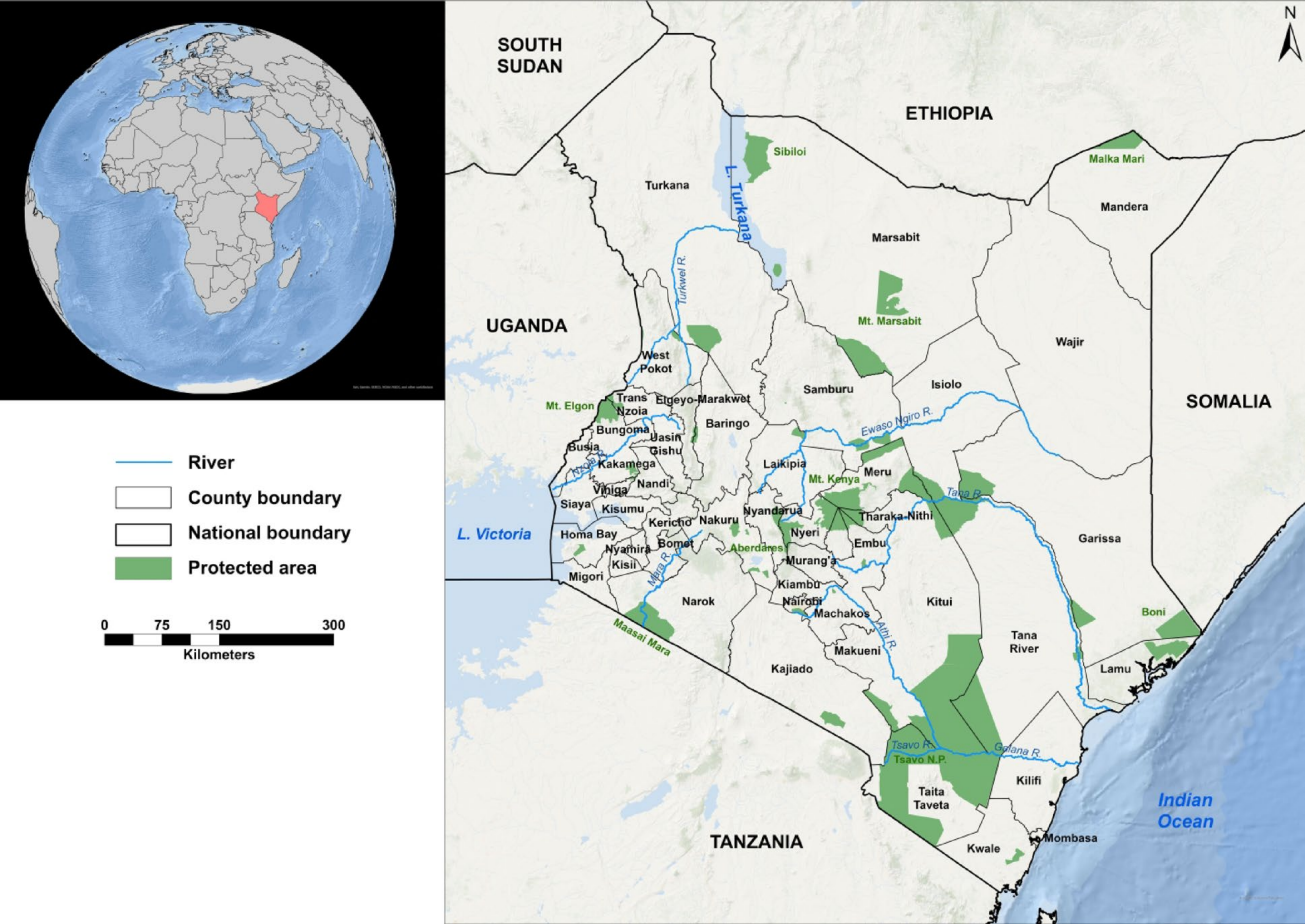
The map of Kenya and its 47 counties

According to the most recent national census conducted in 2019, Kenya had a population of approximately 47.6 million people [23]. By 2025, this figure was projected to reach about 57.5 million and 63 million by 2030, with the population distributed unevenly across the country’s diverse geographical regions [24]. Population density varies considerably across its counties, ranging from under 20 to over 500 people per square kilometre in arid regions and in urban centres respectively. This uneven distribution significantly impacts geographic healthcare accessibility. Since 2013, Kenya has embraced devolution, a system where decision-making and resource allocation for sub-national healthcare services have been transferred to 47 county governments (Fig. 1). This decentralisation aims to address local needs better. However, the national government retains control over major hospitals, health regulations, and overall policy direction.

Kenya’s healthcare system comprises six service delivery levels, ranging from community services (level 1) to national referral hospitals (level 6). It includes primary care at dispensaries and clinics (level 2), health centres (level 3), secondary and tertiary care at sub-county hospitals and medium-sized private hospitals (level 4), primary referral hospitals (level 5), and national referral hospitals (level 6). Facilities are operated by the government, non-governmental organisations (NGOs), faith-based organisations (FBOs), and private sector. Service complexity varies from basic medication and rapid diagnostic tests (RDTs) with lower-skilled staff to advanced laboratory procedures conducted by higher-skilled workers, depending on facility ownership and level.

Kenya has experienced significant disruptions in healthcare services due to health worker strikes. Notably, the prolonged strikes in 2017 markedly impacted service delivery. Another nationwide health workers’ strike occurred from December 2020 to February 2021, driven by demands for better working conditions, including sufficient personal protective equipment, higher risk allowances, and comprehensive health insurance coverage [25, 26]. The duration of the strike varied across different health facilities and categories of workers. Although there is no comprehensive national database tracking these strikes, it is presumed that most health facilities were impacted throughout this period. The 2023/2024 period also witnessed strikes by healthcare workers, further straining the healthcare system [27] and significant delays in recruiting interns, a critical cadre in the provision of healthcare in Kenya [28].

Kenya is highly vulnerable to flooding due to several factors. Situated in the drought-prone greater Horn of Africa region, the country is susceptible to extreme weather events [29]. Heavy rainfall and El Niño cycles often lead to overflowing rivers and flash floods, exacerbated by widespread poverty and inadequate infrastructure, especially in urban areas and in arid and semi-arid regions [30]. In April and May 2024, heavy rains that reached 111% to over 200% of the long-term average across the country [31], caused devastating floods across Kenya, that displaced hundreds of thousands of people, damaged infrastructure, and caused significant loss of life [32]. These recent events underscore the urgent need for improved flood preparedness and mitigation strategies in Kenya.

### Methods overview

A four-step approach was applied. First, data including health facilities (public, private not-for-profit (PfNP), and private for-profit (PfP)), road networks, land use/cover, elevation, population distribution and the flooding extents were assembled. Second, different travel scenarios were defined to account for how flooding affected physical accessibility in Kenya. Third, travel time (TT) from every location to the nearest health facility was estimated for (i) all facilities, (ii) public, (iii) PfP and (iv) PNfP using the least-cost path algorithm while also conducting a sensitivity analysis on the travel speeds and flood extents. Finally, the proportion of population within 30 min, 30–60, 60–90, 90–120 and beyond 120 min TT was extracted, and all estimates were aggregated at subnational units of decision-making (county and sub-county).

### Data

- Healthcare facilities

**Fig. 2:**
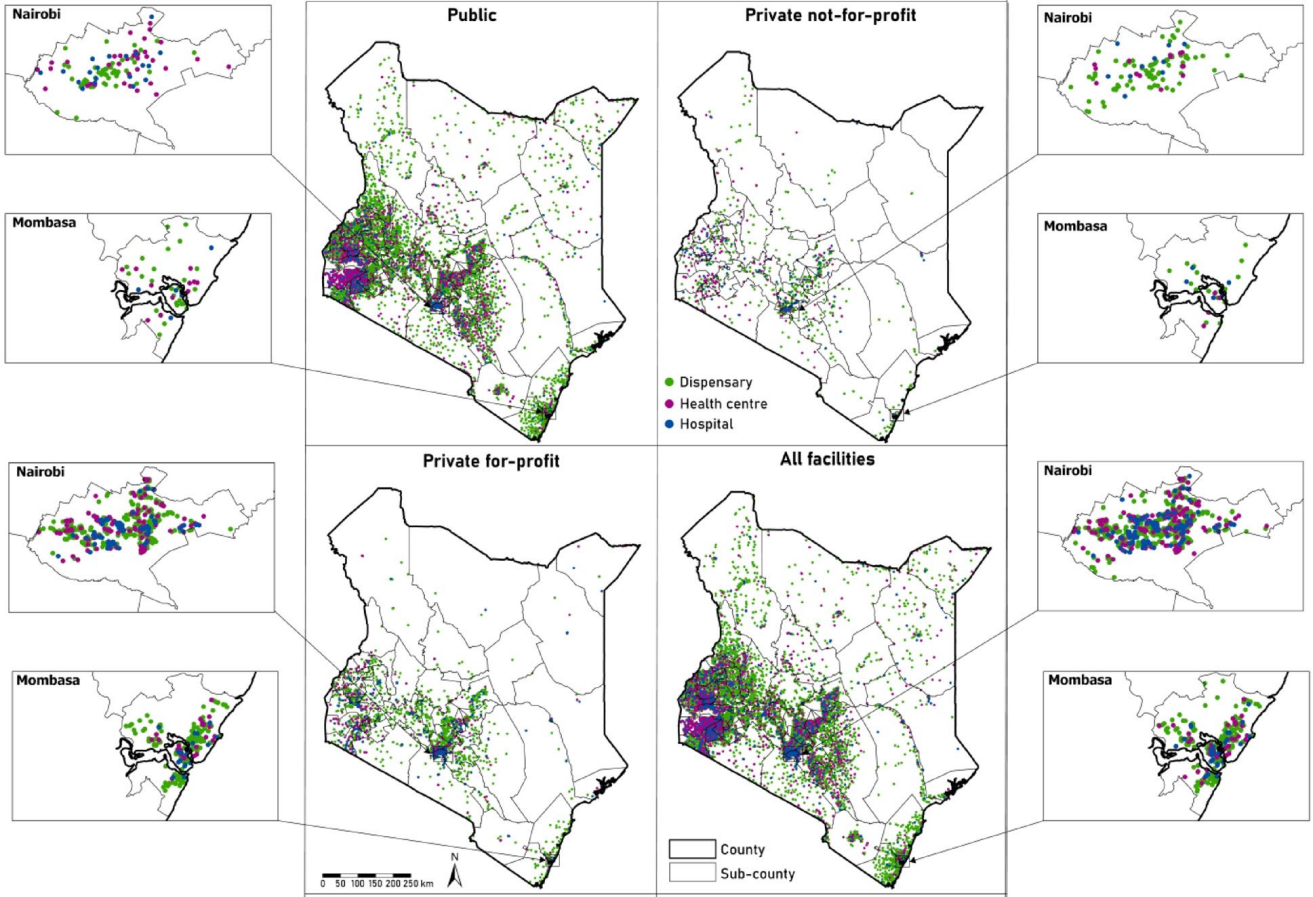
The spatial distribution of health facilities in Kenya by sector in 2023: Public (*n* = 5,586), Private not-for-profit (*n* = 855), private for-profit (*n* = 4,554) and total (both public and private) health facilities (*n* = 10,995)

The Kenya health facility data used in the study were accessed from the Kenya Ministry of Health’s 2023 health facility census that was carried out between the 14th and 29th of August 2023 [33]. The survey aimed to map all health facilities in Kenya including public, private and PNfP (FBOs/NGOs). Some of the information collected during the census and relevant to the current analysis included facility name and code, county, sub-county, ward, latitude, longitude, Kenya Essential Package for Health (KEPH) level and ownership. The focus of the analyses were on all facilities offering general medical services to the public. Facilities offering services to a subset of the population, i.e. company and secondary school clinics, military, police and prison facilities were excluded (*n* = 188). Additionally, we excluded specialised facilities including dental, eye, cancer, tuberculosis, HIV voluntary counselling and testing (VCT) centres, maternal and nursing homes, drop-in centres, mental health centres, hospices, funeral homes, rehabilitation, family planning clinics, radiology/X-ray, blood transfusion, gynaecology, nephrology, kidney dialysis, diagnostic and pharmacies (*n* = 1,192). The final list of facilities contained 10,995 facilities (Fig. 2); 8,112 dispensaries (level 2), 1,995 health centres (level 3) and 888 hospitals (level 4–6).

b. Factors affecting travel to healthcare

Travel time to healthcare facilities was modelled using multiple geospatial factors including road network, land use/land cover (LULC), digital elevation model, water bodies, national parks and population distribution. The road network was classified into primary, secondary, county, and other roads based on the Kenya’s proposed 2023 road register (Supplementary File, FigureS1) [34]. In areas without road network, LULC data derived from 2023 Sentinel-2 imagery at 10 m resolution was used to estimate travel conditions (Supplementary File, Figure S2) [35]. The terrain slope, derived from the Shuttle Radar Topographic Mission (SRTM) at 30-meter resolution digital elevation model (DEM) [36] (Supplementary File, Figure S3), was incorporated to adjust walking and cycling speeds. Additionally, water bodies and national parks [37] were treated as impassable barriers (Fig. 1; Supplementary File, Figure S2). Fine spatial resolution (100 × 100 m) population distribution for the whole country was obtained from WorldPop open spatial demographic data portal [38, 39]. Here, the constrained (population modelled on in areas containing built settlements) gridded population raster for 2023 was used (Supplementary File Figure S4). Additional details of these datasets are provided in Supplementary File.

c. Flooding extent

The flood extents were assessed using high-resolution imagery from the National Oceanic and Atmospheric Administration – Visible Infrared Imaging Radiometer Suite (NOAA-VIIRS) and Sentinel 1 Synthetic Aperture Radar (SAR) sensors, created specifically for flood response efforts in Kenya. The data was sourced from the United Nations Institute for Training and Research (UNITAR) and the International Center for Humanitarian Affairs (ICHA). The ICHA’s Sentinel 1 SAR flood extents data covered flood extents for the entire country from March 31 to May 9, 2024, at a spatial resolution of 30 m, and was created using a change detection model to aggregate all predictions of flood extents since the start of flooding in the country [40]. This was supplemented by flood extents for Tana-Galana River (April 29) based on 0.5 m spatial resolution Pleiades satellite imagery and the Nyando River (May 5) based on Sentinel 1 SAR. Moreover, UNITAR provided two NOAA-VIIRS flood extents at the national level, covering April 24–28 and May 12–16, at a resolution of 375 m [41], which were combined to represent the overall flood extent during that period.

In addition to considering the Sentinel 1 SAR and the NOAA-VIIRS flood extents independently, all datasets were integrated to produce a maximal flooding extent covering the entire country from 31st March to 9th May 2024. Data that were in raster format were first converted to vector format, while those available as images were georeferenced and digitised to extract flood extents. The merged flood extents were then checked for topological errors and polygons that overlapped or shared common boundaries were dissolved into single polygons. All pre-processing steps described above were conducted in ArcGIS Pro version 3.0.3 (ESRI Inc., Redlands, CA, USA).

Finally, to capture the sections of the roads closed due to floods, various newspaper reports and articles were sought out within the flooding period (April-May 2024). Using the *Editor* tool in ArcMap version 10.8.2 (ESRI Inc., Redlands, CA, USA), the sections of the flooded roads were split according to the reports and added an attribute of whether it was flooded or not in the attribute table.

### Travel scenarios

Two main travel scenarios were defined: (i) prior to the flooding (BAU) and (ii) during the flooding, applicable across the entire country. For each scenario, various modes of transport and their corresponding speeds across different road networks and land covers were outlined. In the pre-flooding scenario, a travel framework developed from our earlier national spatial accessibility modelling in Kenya was utilised (5; Table 1). Briefly, this scenario encompasses a hybrid mode of transport involving either walking (areas with no roads), bicycling (lower class roads), motorized (motorbike and vehicles, both public and private in motorable roads) or combined forms of transport based on availability of motorable roads and class of roads.

On the other hand, during flooding, all the roads within the flooding zone were considered impassable (barriers) while the facilities in the flooding zone were deemed non-functional. In addition, parts of the road network outside the flooding zone that the Kenyan government had closed owing to the floods were deemed inaccessible. Outside the flooding zones, we considered reduced speeds (by 50%) in areas across all roads (7; Table 1).

**Table 1:** Travel modes for each road type and land cover category for modelling travel to health facilities before and during flooding)

ID	Category	Mode of transport	Travel Scenarios (km/h)	
			Business-as-usual	During flooding
1	Flooding extents	Various modes and corresponding speeds depending on road type and land cover		Travel barrier
2	Trees	Walking	2.5	1.25
3	Range land	Walking	4.5	2.25
4	Crops	Walking	4	2
5	Bare or built-up	Walking	5	2.5
6	Flooded vegetation	Barrier	0.1	0.05
7	Water	Barrier	0	0
8	Primary road	Motorized	50	25
9	Secondary road	Motorized	30	15
10	County roads	Cycling	10	5 (walk)
11	Other minor roads	Walking	5	2.5

### Estimating travel time

To estimate travel time for the different scenarios, AccessMod version 5.7.17 was used, an open-source tool supported by the World Health Organization (WHO) to analyse geographic accessibility via a least-cost path algorithm [42]. For the BAU scenario, land cover, road network, water bodies and protected areas (usual barriers) were combined via the “merge land cover” module in AccessMod Toolbox to create a merged gridded surface. Based on the resultant single raster, speeds from the BAU model (Table 1) were applied to compute travel time to the nearest facility for (i) all facilities, (ii) public facilities, (iii) PfP facilities and (iv) PNfP facilities (FBOs/NGOs). This was necessary because, during the flooding period, doctors from the public health facilities were on industrial strike [43, 44]. Therefore, clients could only attend either the PfP or PNfP facilities.

Conversely, during flooding, we created a unified gridded surface by utilising land cover, the road network outside the flooding area, and additional barriers (i.e., standard barriers), the maximum flooding extent, and the closed roads beyond the flooding zone. We then assigned speeds to this merged surface to calculate travel time to facilities, categorised into four groups (as previously done for BAU). Further, the travel speeds outside the flooding zones were reduced speeds by 50% (Tables 1 and 7).

For each scenario, the cumulative travel time from every populated location in Kenya based on WorldPop’s population distribution maps was computed towards (anisotropic) the closest facility via the least cost path (cost measured as time) at 100 × 100 m spatial resolution.

### Sensitivity analyses

Two types of sensitivity analyses were conducted. First, in addition to the combined flooding extents, spatial accessibility metrics after flooding based on the individual flooding extents from two sensors (Sentinel 1 SAR and NOAA-VIIRS) were computed. Second, the travel speeds in Table 1 were varied by ± 20% [5, 45], and the least-cost path model was repeated to define an upper and lower bound of travel times for all travel scenarios (BAU and during flooding), facility ownership (all facilities, public, PfP and PNfP and sensor type (Sentinel 1 SAR, NOAA-VIIRS and combined extents).

### Population affected

The geographic coverage estimates (the proportion of the total population within 30 min, between 30 and 60 min, 60–90 min, 90–120 min and beyond 120 min) of the nearest health facility disaggregated by travel scenario, sensor type and level of speed was extracted at national, county and sub-county levels. This was achieved by using *Zonal statistics* tool in ArcGIS Pro Version 3.3.

## Results

### Health facilities assembled

Of the 10,995 facilities assembled for this study over 97% were outside all three flooding zones (99.2%, 97.9% and 97.4% for the NOAA-VIIRS, Sentinel 1 SAR and combined flooding zones respectively). A total of 86, 232 and 289 facilities were identified to be within the NOAA-VIIRS, Sentinel 1 SAR and the combined flooding zones respectivel (Table 2). Majority (68.9%) of the facilities within the three flooding zones were public facilities (45, 172 and 199 facilities for NOAA-VIIRS, Sentinel 2 and combined zones respectively) with the least (4.5%) being PNfP facilities (one, five, and 13 facilities for NOAA-VIIRS, Sentinel 1 SAR and combined zones respectively) Table 2.

**Table 2:** Summary of health facilities by type within each travel time scenario

Type	All facilities	Within flooding zones			Outside the flood zone		
		NOAA-VIIRS	Sentinel 1 SAR	Both	NOAA-VIIRS	Sentinel 1 SAR	Both
Public	5,586	45	172	199	5,541	5,414	5,387
Private not-for-profit	855	1	5	13	854	850	842
Private for-profit	4,554	36	51	77	4,518	4,503	4,477
Total	10,995	86	232	289	10,909	10,763	10,706

### Extent of flooding

**Fig. 3:**
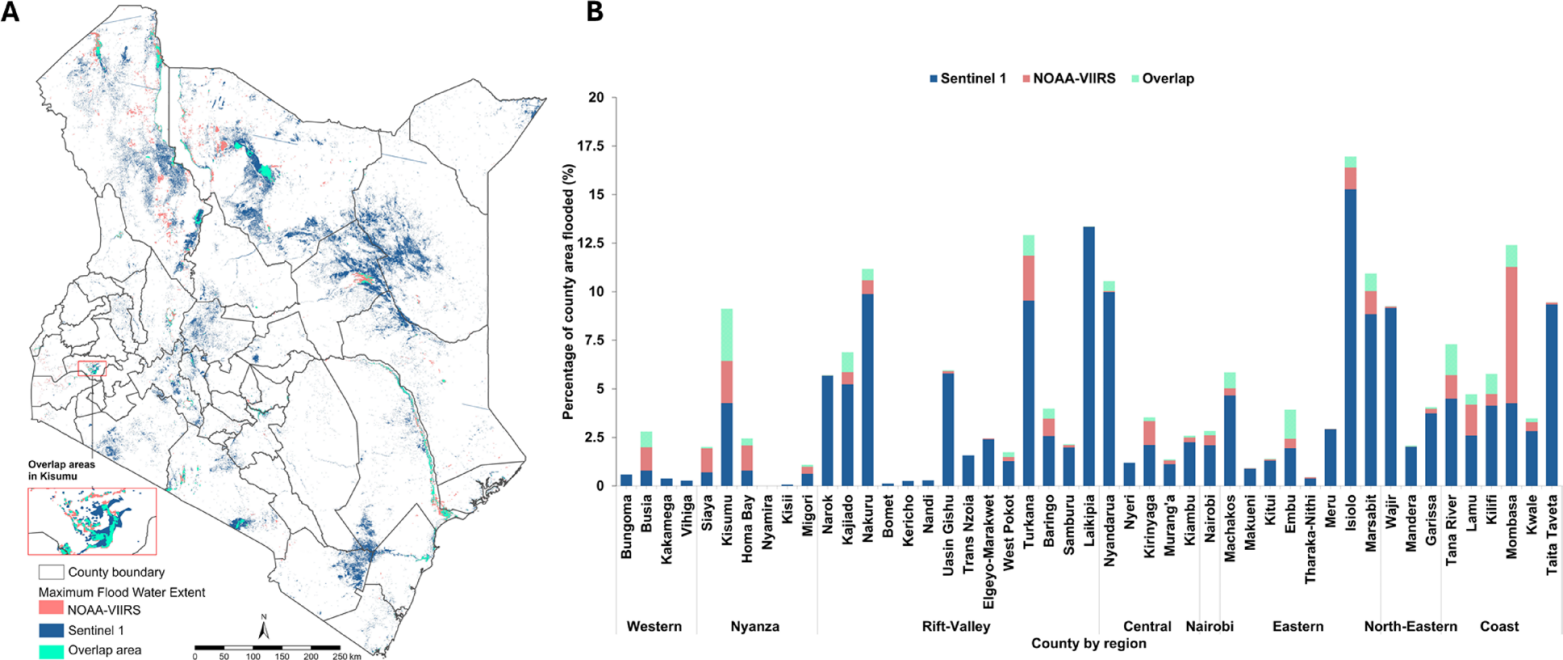
(**A**) The spatial extent of flooding by satellite sensors: NOAA-VIIRS and Sentinel 1 SAR; (**B**) the percentage of flooded regions that are covered by both Sentinel 1 SAR and NOAA-VIIRS that do not overlap and those that overlap across counties

The total flood-affected area was 43,422.9 km², representing 7.3% of Kenya’s land area. This was mainly due to Sentinel 1 SAR, which covered 39,115.3 km² (6.6%), compared to NOAA-VIIRS, which covered 7,345.2 km² (1.2%). The overlap in sensor detection was 3,037.6 km², or 0.5% of the total area, and 7.0% of the total flood extent area (Fig. 3A, Supplementary File, Figure S5). Nyamira was the only county with no flood extents recorded from either sensor. Nine counties (Trans Nzoia, Nyeri, Bungoma, Kakamega, Nandi, Vihiga, Kericho, Bomet, and Kisii) were not included in NOAA-VIIRS flood extents but appeared in Sentinel 1 SAR data. Isiolo showed the highest proportion of flooded area at 17.0% based on maximum extents and also had the highest non-overlapping flood extents at 15.3%.

### Population within flooding zones

An estimated 1,734,531 people, representing 3.1% of Kenya’s population, were located within these maximum flood extents. By satellite, an estimated 1,360,396 people were located within the S1-defined extents while 488,530 people were located within the NOAA-VIIRS-defined extents. By county, people residing in Garissa county were the most affected by the floods according to the combined and the S1 defined extents with estimated 155,162 and 150,013 people respectively. However, from the NOAA-VIIRS-defined extents, Mombasa county was the most affected with estimated 87,273 residing within the flooded extents (Supplementary file, Table S1).

### Travel time to health facilities pre- and post-flooding

#### Pre flooding

**Fig. 4:**
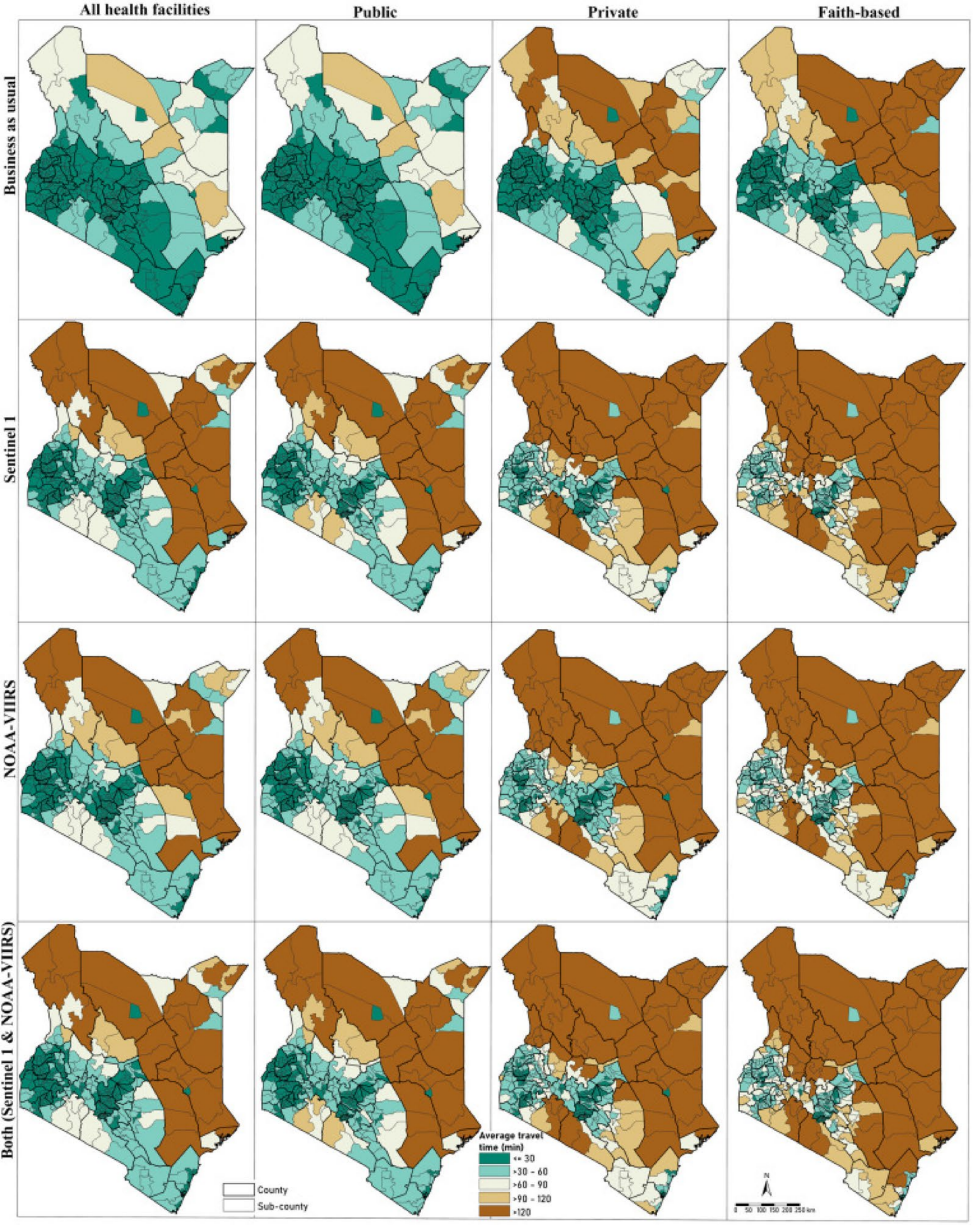
Average travel time to the nearest health facility at sub-county level by facility type (all, public, PfP and PNfP) for the BAU and worst-case scenarios (Sentinel 1 SAR, NOAA-VIIRS and the combined flooded extents)

Under the BAU scenario, the national average travel time to the closest facility was 19.6 min (range: 16.4–24.4). When disaggregated by facility type, the average travel times were 20.7 min (17.3–25.7) for public facilities, 37.8 min (31.6–47.1) for PfP facilities, and 49.2 min (41.1–61.4) for PNfP facilities. Nairobi County had the shortest mean travel times: 2.5 min (2.2–3.1) overall, 3.8 min (3.3–4.7) for public facilities, 2.9 min (2.5–3.5) for private facilities, and 4.1 min (3.5–4.9) for PNfP facilities (Fig. 4). In contrast, Marsabit County recorded the longest average travel times across all types of health facilities: 76.3 min (63.7–95.3) for all facilities, 77.1 min (64.3–96.2) for public facilities, and 185.4 min (154.6–231.6) for private facilities (Supplementary File, Figures S6-S8). Mandera county, however, showed the highest average travel time for PNfP facilities at 363.0 min (302.6–453.7) (Supplementary File, Figure S9). Thirteen counties (Garissa, Isiolo, Kajiado, Kitui, Lamu, Mandera, Marsabit, Narok, Samburu, Tana River, Turkana, Wajir and West Pokot) had travel times longer than the national average travel times for all, public, and private facilities (Supplementary File, Figures S6-S8), while twelve counties surpassed the national average travel time for PNfP facilities (Supplementary File, Figure S9).

### Post flooding

In all worst-case scenarios (NOAA-VIIRS, Sentinel 1 SAR, and the combined data), the national average travel time to health facilities exceeded that of the BAU scenario (Fig. 4). Specifically, the average travel time to the nearest facility (covering all facilities) was 55.6 min (range: 46.5–69.6) for Sentinel 1 SAR-derived extents, 45.8 min (38.3–57.1) for NOAA-VIIRS, and 56.3 min (47.0–70.3) for the combined flooded extents. Similar patterns were observed across the different facility ownership types and flooding scenarios, with the longest travel times for PNfPs facilities was 113.5 min (range: 94.6–191.5) and the shortest for public facilities was 48.5 min (range: 40.5–74.5), regardless of flooding extent. The combined flooding scenario resulted in the greatest reduction in travel times (Fig. 4; Supplementary Figures S6-S9).

### Geographic coverage pre- and post-floods

#### Pre flooding

**Fig. 5:**
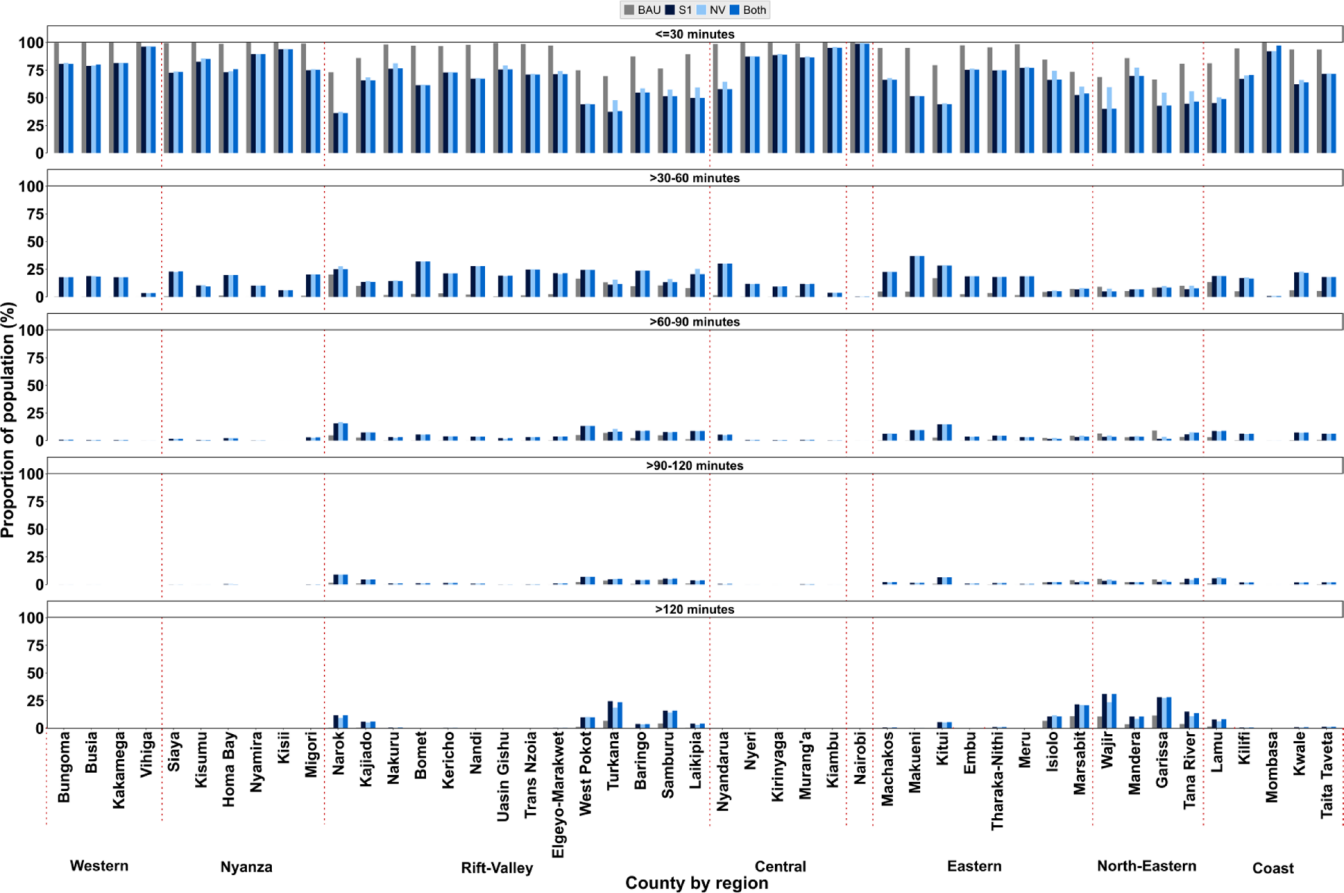
Proportion of population within 30-minute travel time bands for all facilities per scenario (BAU, Sentinel 1 SAR, NOAA-VIIRS and combined flooded extents)

Before the floods (BAU), 94.0% of the population had access to health facilities within 30 min (Fig. 5, panel 1) and 0.7% lived outside 2 h travel time (Fig. 5, panels 2–5). County level population access within 30-min access varied between 66.4% (Garissa) to 100% (Kisii). Overall, 30 of 47 counties had over 94% of their population within the 30-minute access range, matching the national average. Only eight counties had their entire population within 60 min, 16 counties within 90 min and 20 counties within 2 h travel time.

### Post flooding

During flooding events (under combined scenarios), the proportion of the population with timely access (within 30 min) decreased from 94.0% to 73.3%. Specifically, between sensors, only 19 counties (Sentinel 1 SAR scenario), 20 counties (NOAA-VIIRS), and 17 counties (combined) maintained at least 75% of their population coverage within 30 min, as shown in Fig. 5, panel 1.

The reduction in population coverage within this 30-minute threshold at the county level ranged from as low as 1.0% in Nairobi to as high as 51.0% in Narok. The most significant declines (41.0–51.0%) were seen in Narok, Turkana, Makueni, Tana River, Kitui, Laikipia, Lamu, Wajir, Nyandarua, and West Pokot. Conversely, counties such as Nairobi, Vihiga, Kiambu, Kisii, Mombasa, and Nyamira experienced relatively small coverage losses (1–10%; Fig. 5). Only five counties- Kakamega, Kisii, Nairobi, Nyamira, and Vihiga- retained full population access within a 2-hour travel time. Counties including Wajir, Garissa, Turkana, Marsabit, Samburu, Isiolo, and Tana River saw 15–31% of their populations falling beyond 2 h of access after the floods, an increase from 4 to 12% before the floods (Fig. 5, panel 5). Similar reductions in access to health services across public health facilities were observed, with the most severe impacts in counties in northern and northeastern Kenya, which have fewer private and PNfP facilities (Supplementary Files, Figures S10-S12).

## Discussion

The 2024 Kenya floods recovery needs assessment and other humanitarian impact reports highlighted extensive disruptions to health services following the March–May 2024 floods [16]. While these reports documented cross-sectoral impacts and proposed broad mitigation measures, they did not quantify health service coverage losses. Our study addresses this critical gap by assessing the impact of floods on geographical access to health care facilities by quantifying travel time during pre-floods (BAU) and post-floods. The post-floods scenarios were analysed using Sentinel 1 SAR and NOAA-VIIRS satellite data and a combined scenario integrating the two datasets. Our results reveal a substantial reduction in timely (within 30-min) access to health care facilities, with population coverage dropping from 94% (52,488,181) pre-flood to 73% (40,916,717) post-floods. The increased travel time to health facilities was also observed across all health facility types. These findings underscore the sensitivity of Kenya’s health system to extreme weather events and highlight the urgent need for frameworks that aid in disaster preparedness to mitigate the risk of loss of access to healthcare during crises [46].

Flooded extents varied substantially across counties (Supplementary Figure S13) reflecting a complex interplay of topographical and socio-environmental factors beyond rainfall intensity [47]. For instance, the largest flood extents were observed in diverse ecological and geographic settings: semi-arid counties such as Isiolo, Laikipia, and Turkana, which typically experience low rainfall and limited drainage infrastructure; Mombasa, a coastal urban county prone to tidal influences and poor urban drainage; and Nakuru, a highland region with urban zones where topography and land use may contribute to localized flooding. These spatial differences were further influenced by the characteristics of specific satellite sensors. For example, Sentinel-1 SAR, with its finer spatial resolution (5 × 20 m), was able to detect small-scale flooded areas across all counties that may have been missed by NOAA-VIIRS, likely due to its coarser resolution (375 m). In addition, Sentinel-1 SAR’s advantages include (i) the sensor produces microwaves which have the ability to penetrate through clouds, whereas optical sensors do not have the ability to see through clouds (ii) the SAR is an active sensor, which means that it produces its own electromagnetic radiation in this case microwaves, and does not rely on sunlight hence can detect floods during the day and night, whereas NOAA-VIIRS is an optical sensor and relies on sunlight for its sensing and (iii) has a shorter revisit period of six days as compared to 16 days for NOAA-VIIRS. Also, floods extents captured by SAR covered between 31st March–9th May 2024 while for NOAA-VIIRS, the flooded extents covered between 24th−28th April and 12th−16th May 2024. Notably, in Mombasa County NOAA-VIIRS detected wider flooding extents than Sentinel 1 SAR, while in counties like Narok, Nakuru and Laikipia, the opposite was true. This sensitivity of results and flood extent estimates to the satellite sensor selected highlights the crucial role of high-resolution, real-time geospatial data in disaster response efforts [48]. In Nyamira County, neither sensor detected flood extents despite reports of infrastructural damages caused by the heavy rainfall [49, 50]. Such limitations in satellite-based flood monitoring emphasise the need for standardized, multi-source flood assessment methods to enhance detection accuracy, especially in low-resource settings [6, 51].

Under the BAU scenario, over 90% of the population resided within half an hour of a health facility. However, this national coverage masks out some of the huge inequalities particularly in arid and semi-arid counties such as Garissa, Isiolo, Marsabit and Wajir where travel times exceeded 1 h, reflecting persistent access challenges in marginalised regions [2, 45]. The floods further exacerbated these disparities reducing the coverage of people within 30 min nationally to 73% (combined scenario), with northeastern counties experiencing longer travel times above national average. These counties are characterised by limited road infrastructure, and they heavily rely on public facilities. For instance, Mandera had an average travel time of 37 min to a public facility under the BAU scenario as compared to over 3.5 h average travel time to PfP and PNfP facilities. After floods travel time to PNfP facilities alone in this county doubled (6 h). In addition, PfP and PNfP facilities that serve as critical alternatives during public sector strikes [27, 43], were concentrated in urban centres, hence shorter travel times leaving rural populations with even fewer options post-disaster (Fig. 4).

Floods have disproportionately disrupted timely access to health facilities, exposing critical geographic, hydrological, and infrastructural vulnerabilities across counties [47, 52]. Counties with the greatest loss in 30-minute coverage, such as Narok, Tana River, Lamu, Kitui, and Makueni, are located in major flood-prone areas identified in flood advisories issued months before the floods [53, 54]. Notably, Kitui experienced the highest coverage loss at 51% and also sustained the most extensive damage to health facilities at 47%, including a referral hospital [16]. Turkana, Tana River, Wajir, and Lamu had the highest proportion of facilities within flooded areas, with at least nine facilities in Tana River reported damaged [19]. Laikipia and Nyandarua were among those with the largest flooded extents. The correlation between health facility exposure and coverage decline was also evident in longer travel times (60 to 120 min) across counties like Marsabit, Turkana, Tana River, Wajir, Isiolo, and Garissa, where limited road infrastructure further hindered access.

Our findings reveal that even minor disruptions in coverage (1–10%) in densely populated counties such as Vihiga, Kiambu, Kisii, Mombasa, and Nyamira can lead to significant increases in the number of people facing longer travel times, despite generally good infrastructure. These delays have varied impacts on health service utilization. Notably, Kiambu experienced some of the steepest declines in hospital admissions, outpatient visits, and immunization services [16]. This underscores the need to interpret coverage losses not just in percentage terms but through the lens of absolute population affected and spatial context. The fact that only five counties had the entire population within 2 h travel time post-floods down from 20 pre-floods, further underscores the urgency of integrating disaster preparedness policies to ensure responsive, equitable healthcare access during extreme weather events [4].

These vulnerabilities exposed by floods are further compounded by systemic challenges such as the recurrent health workers’ strike [26, 27, 43, 55]. The 2017 national wide strike disrupted health service delivery for about 300 days, and illustrates how prolonged and destabilizing such labour disputes can be [56]. The 2024 strike coincided with the national flood emergency, further constraining the system’s ability to respond [57]. Public health facilities are disproportionately affected during such periods, often scaling down operations or temporarily closing [57, 58]. This further limit access for communities already facing reduced geographic coverage due to flood‑related disruptions. As a result, patients are forced to delay treatment or seek care from private facilities that are more expensive and unevenly distributed, leading to longer travel times, higher out‑of‑pocket costs, worsening health conditions, and in some cases preventable loss of life [58]. This convergence of climate‑related and labour‑related disruptions deepens existing inequities and erodes the overall resilience of the health system during crises.

### Policy implications

Kenya continues to experience recurrent and severe flood events resulting in fatalities, destruction and disruption of essential infrastructural services, including health care [59]. Our study highlights the sensitivity of Kenya’s health system to flood-related shocks, offering insights that complement existing recovery strategies and help mitigate long-term impacts on the population. Policymakers should prioritise resource allocation to high-risk counties, not only those vulnerable to flooding but also those susceptible to post-disaster health risks. For instance, Tana River, Lamu and Siaya counties experienced cholera outbreaks following the March-May floods. Mapping access to care losses including at the sub-county level (Fig. 3) offers actionable insights for targeted emergency response. Further, leveraging multi-source flood assessment approaches, including geospatial and drone technologies [60] and including participatory approaches to gather historical flood impacts from communities, can enhance informed decision-making and develop contingency strategies, such as mobile clinics and emergency transport corridors, to buffer access disruptions during disasters. Enhancing health system resilience amid escalating climate risks will require stronger intersectoral coordination among health, infrastructure, humanitarian, and meteorological agencies. It is also critical that both national and county governments treat flood advisories and weather forecasts [53, 61] as actionable early warnings, prompting proactive disaster prevention and preparedness rather than reactive responses.

### Strengths

This study offers several strengths. Unlike previous similar studies [62, 63] that rely solely on assumptions such as reduced travel speeds during flood events, we incorporated actual flooded extents to comprehensively capture access disruptions caused by flooding. To overcome the limitations of any single sensor, we employed multi-sensor data. Further, the Sentinel 1-derived flood extents was an aggregation of satellite data since the flooding started and hence not from a single point in time. In addition, Sentinel 1 SAR sensor is not affected by cloud cover and by lack of daylight which ensured uninterrupted monitoring of flooding events. We also integrated official records on road closures provided by Kenyan road authorities [18, 64]. Additionally, since travel speeds used in the analysis are estimates rather than directly observed, we conducted a sensitivity analysis increasing/decreasing speed by 20% to adjust for uncertainties. We also used the most recent and comprehensive database of health facilities, compiled through a nationwide survey conducted by the Ministry of Health. Finally, we disaggregated our analysis by health sector owing to doctor strike and household wealth differentials in terms of money that influence the ability to access care.

#### Limitations

Notwithstanding the valuable insights on understanding the impacts of flooding on access to health care in Kenya, there are several limitations. The change detection model that produced the Sentinel 1 SAR-defined flood extents may have resulted to some false positives within the semi-arid areas for instance in Wajir. NOAA-VIIRS is an optical sensor, and its flood detection capabilities is constrained by cloud cover and lack of daylight, further contributing to potential underestimation of inundation. This study incorporated flooded road segments in addition to the flood extents, but only segments reported to have flooded were accounted for possibly missing other affected roads not documented. Furthermore, we did not know the extent of damaged roads, limiting our mapping of infrastructure disruption. We assumed that population within flood zones had no alternative means of accessing health facilities. However, in some affected areas boats were reportedly used. We also lacked data on conditions outside the mapped flood zones, so we couldn’t account for disruptions caused by unusually heavy rainfall in those areas. We assumed individuals travel to the nearest facility, which may not reflect actual healthcare-seeking behaviour or preferences. All flooded roads were assumed impassable although some vehicles (e.g. trucks) may have been able to navigate certain segments. Lastly, this study only focused on quantifying the impact of floods on geographical access to health facilities, highlighting spatial disparities in health care access during floods. However, further research is needed to assess county-level adaptive capacity which is essential for understanding overall health system vulnerabilities to extreme weather events.

## Conclusions

One of the core responsibilities of the health system is to prevent, prepare for, detect and respond to public health threats [65] including floods, the second most prevalent natural disaster in Kenya. Our findings show that flood events significantly reduce geographical access to healthcare services, with pronounced subnational disparities across flood-prone counties, semi-arid regions with persistent infrastructural deficits, and densely populated regions. The loss of access to health care is largely driven by impacts on flood-exposed health facilities, spatial extent of flooding, and damaged transport system inhibiting access. However, the impact on health services utilisation varies sub-nationally. Considering compounding health risks associated with floods such physical injuries, the spread of communicable diseases (e.g., measles) due to overcrowding in displacement settlements, disease outbreaks (malaria, cholera, etc.), it is critical to ensure that health care system can sustain service delivery amid recurring climate shocks.

## Supplementary Information

Supplementary Material 1

## Data Availability

The health facility data is available from the Ministry of Health (https://www.health.go.ke/contact-us).Sentinel-2 flooding extent from the International Center for Humanitarian Affairs (ICHA; https://redcrosske.maps.arcgis.com/home/item.html?id=bce5234218c547439ce2a71f7cbeb4e2) and NOAA-VIIRS flooding extent from the United Nations Institute for Training and Research (https://unosat.org/products/). The roads data from the Kenya Roads Board portal (https://maps.krb.go.ke/kenya-roads-board12769/maps). The Digital elevation model can be downloaded from the Regional Centre for Mapping of Resources for Development (RCMRD) geoportal (https://gmesgeoportal.rcmrd.org/datasets/rcmrd::kenya-srtm-dem-30meters/about). The land use land cover from the Environmental Systems Research Institute (ESRI’s) ArcGIS platform (https://www.arcgis.com/home/item.html?id=6df9ed7a1eda4ed58023456b7c5484fd), protected areas for the Global database of protected areas (https://www.protectedplanet.net/en/about) and population data from the WorldPop open spatial demographic data portal (https://www.worldpop.org/). **.**.

## Abbreviations

BAU: Business-as-usual
DEM: Digital elevation model
ESRI: Environmental systems research institute
FBOs: Faith-based organisations
ICHA: International center for humanitarian affairs
KEPH: Kenya essential package for health
LULC: Land use/land cover
MoH: Ministry of health
NGOs: Non-government organisations
NOAA-VIIRS: National oceanic and atmospheric administration – visible infrared imaging radiometer suite
PfP: Private-for-profit
PNfP: Private-not-for-profit
RDTs: Rapid diagnostic tests
SAR: Synthetic aperture radar
SRTM: Shuttle radar topographic mission
SSA: Sub-Saharan Africa
TT: Travel time
UNITAR: United nations institute for training and research
VCT: Voluntary counselling and testing
WHO: World health organization
