# Supplementary material for: Impact analysis of flood-induced changes in geographical accessibility and coverage to healthcare in both public and private sector, 2024, Kenya
Source: Int J Health Geogr. 2026 Mar 18;25:25. doi: 10.1186/s12942-026-00461-x (PMC13122988; doi:10.1186/s12942-026-00461-x)
Supplement: Supplementary file 1 — Supplementary Material 1 [file 12942_2026_461_MOESM1_ESM.docx]

**Supplementary File**

# **Data**

Factors affecting travel to healthcare are described below:

***Road network:*** The road network was based on the roads inventory and conditions data collected by Kenya Roads Board through a national survey conducted in 2018 (1) . The roads are classified to reflect the function served by the roads and have undergone several revisions since Kenya's road network was gazetted in January 2016. The classification adopted in this study is based on the proposed 2023 road register, containing recent proposed road classification changes (1). The roads were then reclassified as primary (international and national trunk roads), secondary, county, and others (2,3) as shown in Figure S1.

***Land use/cover:*** In areas not covered by road network and between roads, we used land use/cover (LULC) data. The LULC was derived from ESA Sentinel-2 imagery at 10m resolution for the year 2023 based on a deep-learning AI land classification model trained with human-labelled image pixels (4).The algorithm generated LULC predictions for eight classes in Kenya including rangeland (open areas with homogenous grasses with little to no taller vegetation), bare ground (areas of rock, soil, sand or deserts with very sparse to no vegetation), built up area (human made structures, major road and rail networks; large homogenous impervious surfaces), crops (human planted cereals, grasses, and crops not at tree height), flooded vegetation (vegetated areas with intermixing of water throughout a majority of the year or seasonally flooded), trees (significant clustering of tall dense vegetation), snow/ice (large homogenous areas of permanent snow or ice, often in mountain areas) and water (areas where water is predominantly present throughout the year). The LULC map is shown in Figure S2.

***Digital elevation model:*** The walking or bicycling speeds are modified by the topography of the terrain. To account for this, we utilised slope based on the digital elevation model (DEM). The DEM was obtained from the Shuttle Radar Topographic Mission (SRTM) at 30 x 30m spatial resolution accessible through the Regional Centre for Mapping of Resources for Development (RCMRD) geoportal (5). The elevation map is shown in Figure S3.

***Water bodies and national parks:*** Water bodies and national parks were considered as transport barriers (impassable). The water bodies were derived from the land use land cover surface (Figure S2), while the national parks were based on the global database of protected areas (6) as shown in Figure 1.

***Population distribution data:*** The constrained (population modelled on in areas containing built settlements) gridded population raster for 2023 sourced from WorldPop open spatial demographic data portal (7) is shown in Figure S4.


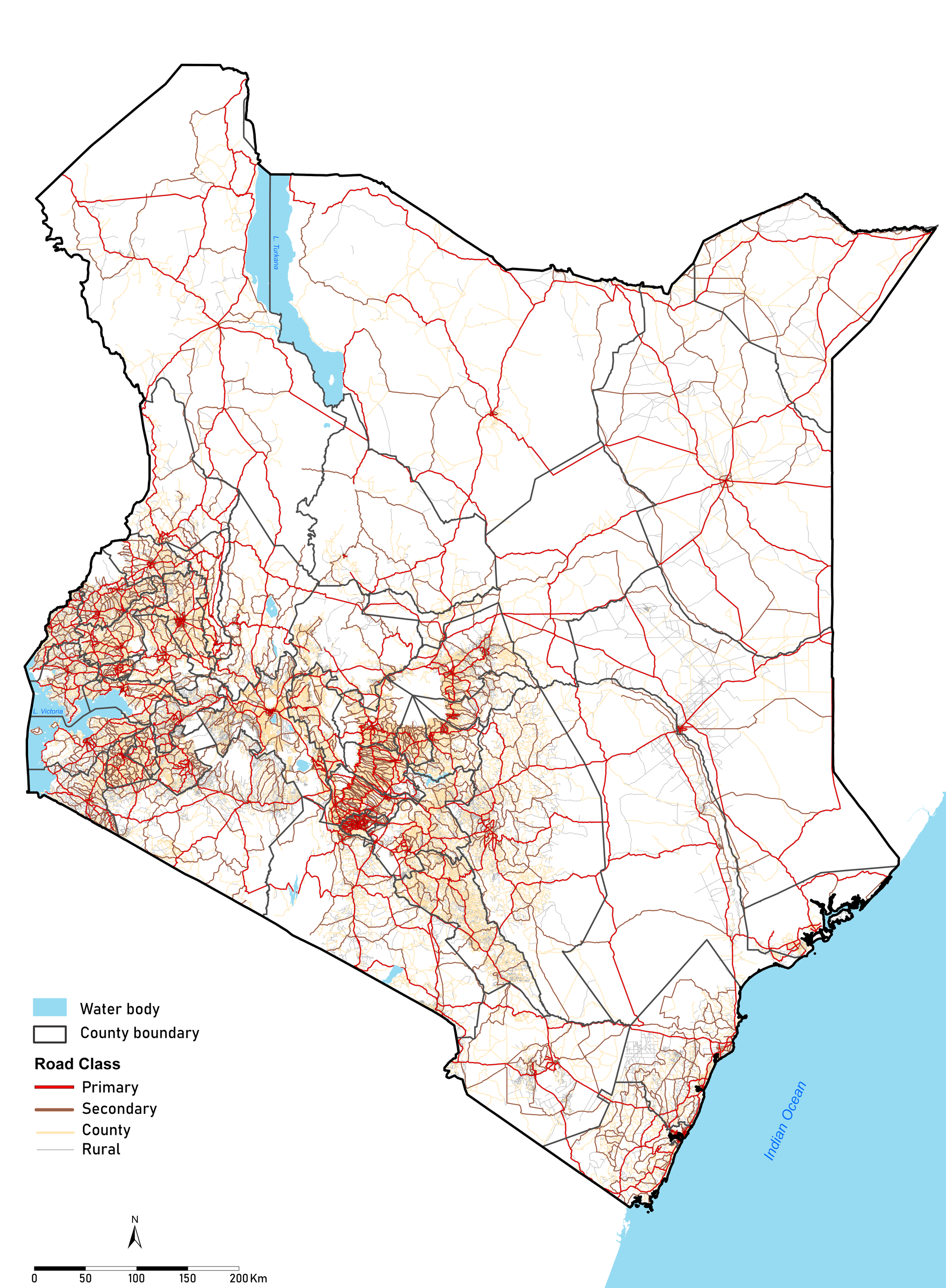


**Figure S1:** Map showing the road network of Kenya


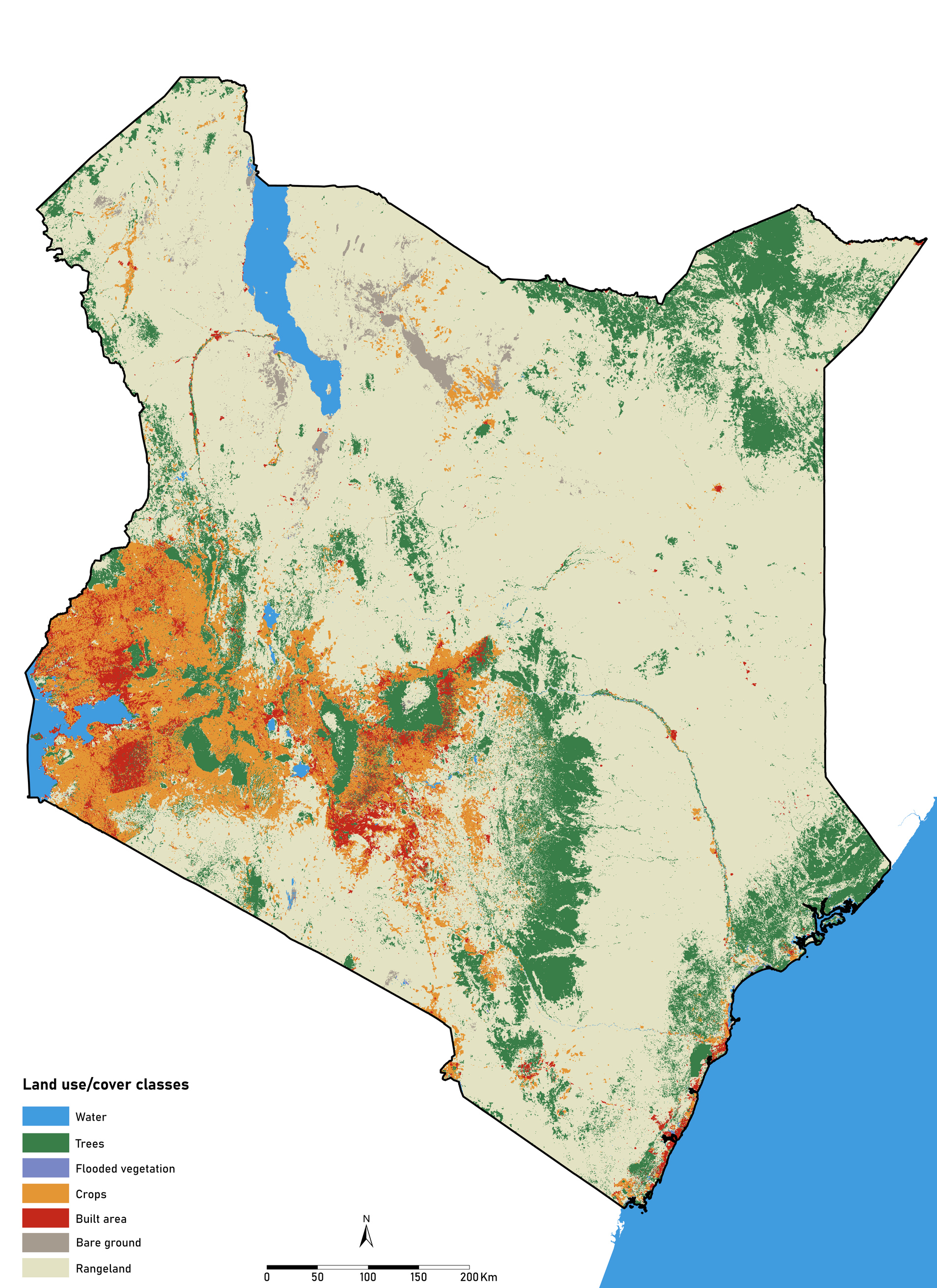


**Figure S2:** Land use/cover classes across Kenya.


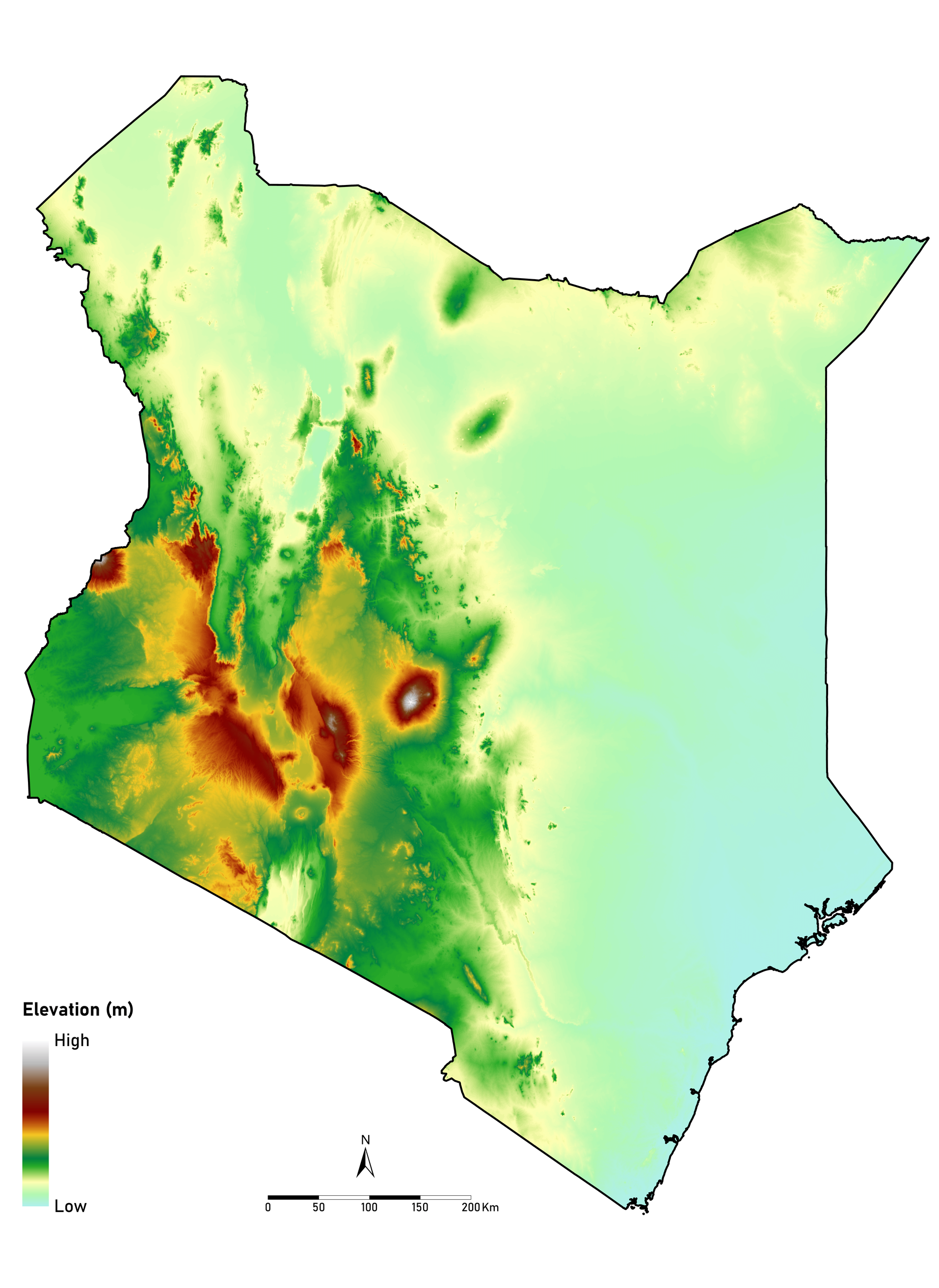


**Figure S3:** Elevation across Kenya.


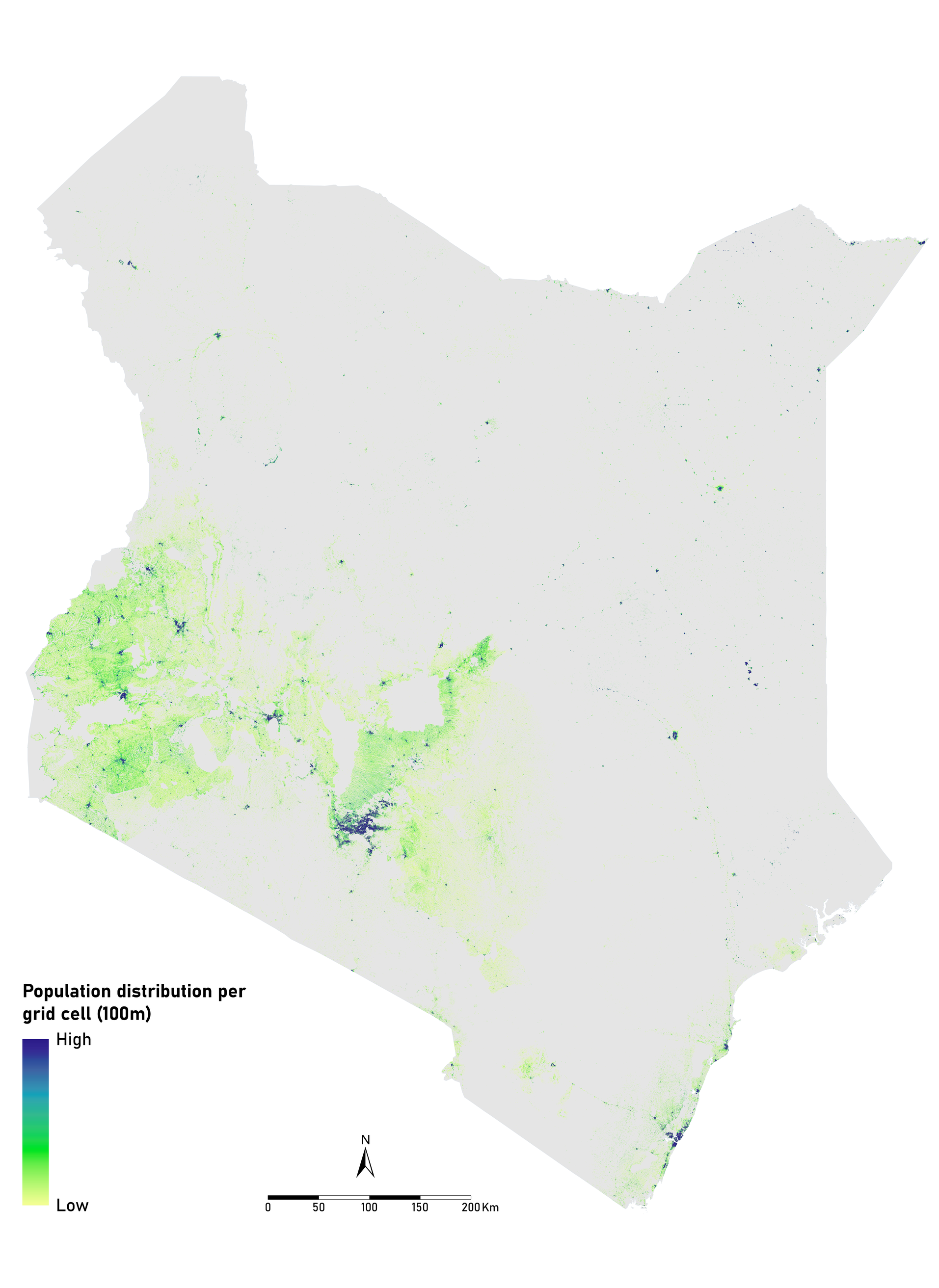


**Figure S4:** Population distribution (modelled in areas containing built settlements) across Kenya.

1. **Flood extent area in square kilometres**


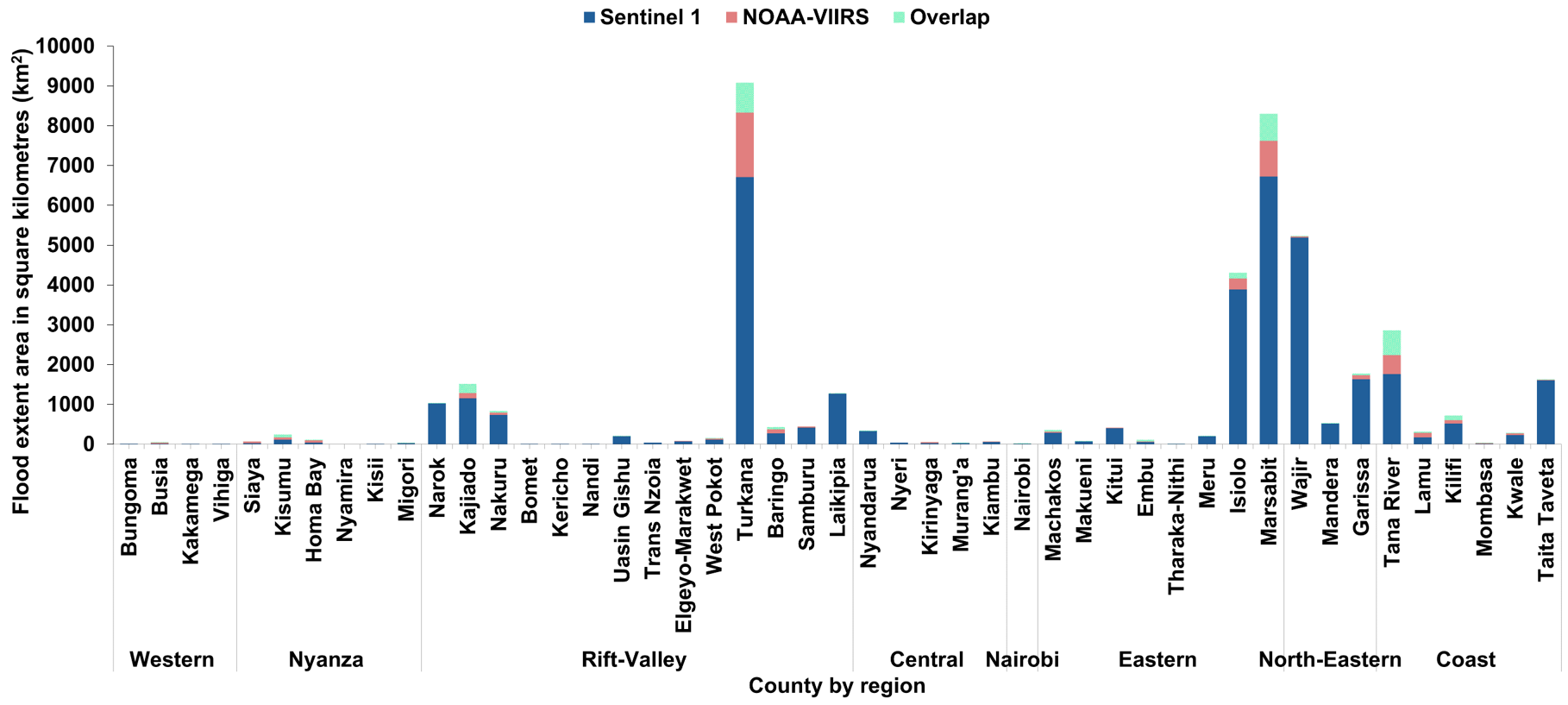


**Figure S5:** Boxplots at county level by scenario (lower average and upper bounds) for all facilities and average national travel time.

**Table S1:** Population affected by floods per county by flooding extent.

| **County** | **Sentinel 1** | **NOAA-VIIRS** | **Combined extents** |
| --- | --- | --- | --- |
| Baringo | 32,198 | 9,547 | 35,694 |
| Bomet | 696 | 0 | 696 |
| Bungoma | 9,082 | 0 | 9,082 |
| Busia | 5,967 | 12,850 | 15,908 |
| Elgeyo-Marakwet | 10,231 | 343 | 10,575 |
| Embu | 7,681 | 3,492 | 9,324 |
| Garissa | 150,013 | 12,200 | 155,163 |
| Homa Bay | 18,135 | 38,953 | 50,750 |
| Isiolo | 38,481 | 11,030 | 43,086 |
| Kajiado | 33,054 | 3,749 | 35,134 |
| Kakamega | 2,582 | 0 | 2,582 |
| Kericho | 1,206 | 0 | 1,206 |
| Kiambu | 14,538 | 16,025 | 30,101 |
| Kilifi | 53,950 | 51,143 | 95,751 |
| Kirinyaga | 6,097 | 4,170 | 10,076 |
| Kisii | 1,193 | 0 | 1,193 |
| Kisumu | 57,232 | 40,875 | 78,166 |
| Kitui | 10,337 | 639 | 10,829 |

**Table S1 continued…**

| **County** | **Sentinel 1** | **NOAA-VIIRS** | **Combined extents** |
| --- | --- | --- | --- |
| Kwale | 39,494 | 13,427 | 52,245 |
| Laikipia | 71,381 | 108 | 71,444 |
| Lamu | 11,679 | 12,428 | 20,563 |
| Machakos | 24,502 | 5,928 | 26,795 |
| Makueni | 3,248 | 71 | 3,301 |
| Mandera | 71,188 | 46 | 71,191 |
| Marsabit | 55,466 | 9,996 | 63,880 |
| Meru | 9,489 | 239 | 9,688 |
| Migori | 5,616 | 6,109 | 11,233 |
| Mombasa | 3,364 | 87,274 | 89,770 |
| Murang'a | 5,319 | 544 | 5,807 |
| Nairobi | 24,627 | 61,517 | 83,137 |
| Nakuru | 91,688 | 8,435 | 97,251 |
| Nandi | 1,720 | 0 | 1,720 |
| Narok | 32,763 | 224 | 32,790 |
| Nyamira | 0 | 0 | 0 |
| Nyandarua | 39,773 | 602 | 39,859 |
| Nyeri | 792 | 0 | 792 |
| Samburu | 21,001 | 72 | 21,055 |
| Siaya | 7,560 | 10,506 | 17,021 |
| Taita Taveta | 1,537 | 113 | 1,626 |
| Tana River | 72,860 | 46,566 | 91,214 |
| Tharaka-Nithi | 644 | 124 | 768 |
| Trans Nzoia | 7,075 | 0 | 7,075 |
| Turkana | 119,634 | 17,607 | 132,350 |
| Uasin Gishu | 32,380 | 1,093 | 33,438 |
| Vihiga | 1,738 | 0 | 1,738 |
| Wajir | 141,824 | 92 | 141,893 |
| West Pokot | 9,361 | 393 | 9,571 |
| **Total** | **1,360,396** | **488,530** | **1,734,531** |

# **Access to health facilities pre- and post-flooding by facility types across scenarios.**

***All Health Facilities***

Figure S6 is a boxplot showing the average national travel time including lower average and upper bounds at county level by scenario (Business-as-usual (BAU), Sentinel 1 SAR (S1), NOAA-VIIRS (NV) and Both sensors worst case scenario (WCS)) for all health facilities. Other similar box plots disaggregated by facility types (public, private-for-profit and private-for-not-for-profit (faith-based and non-governmental) as shown in Figures S7-S10 are discussed below.


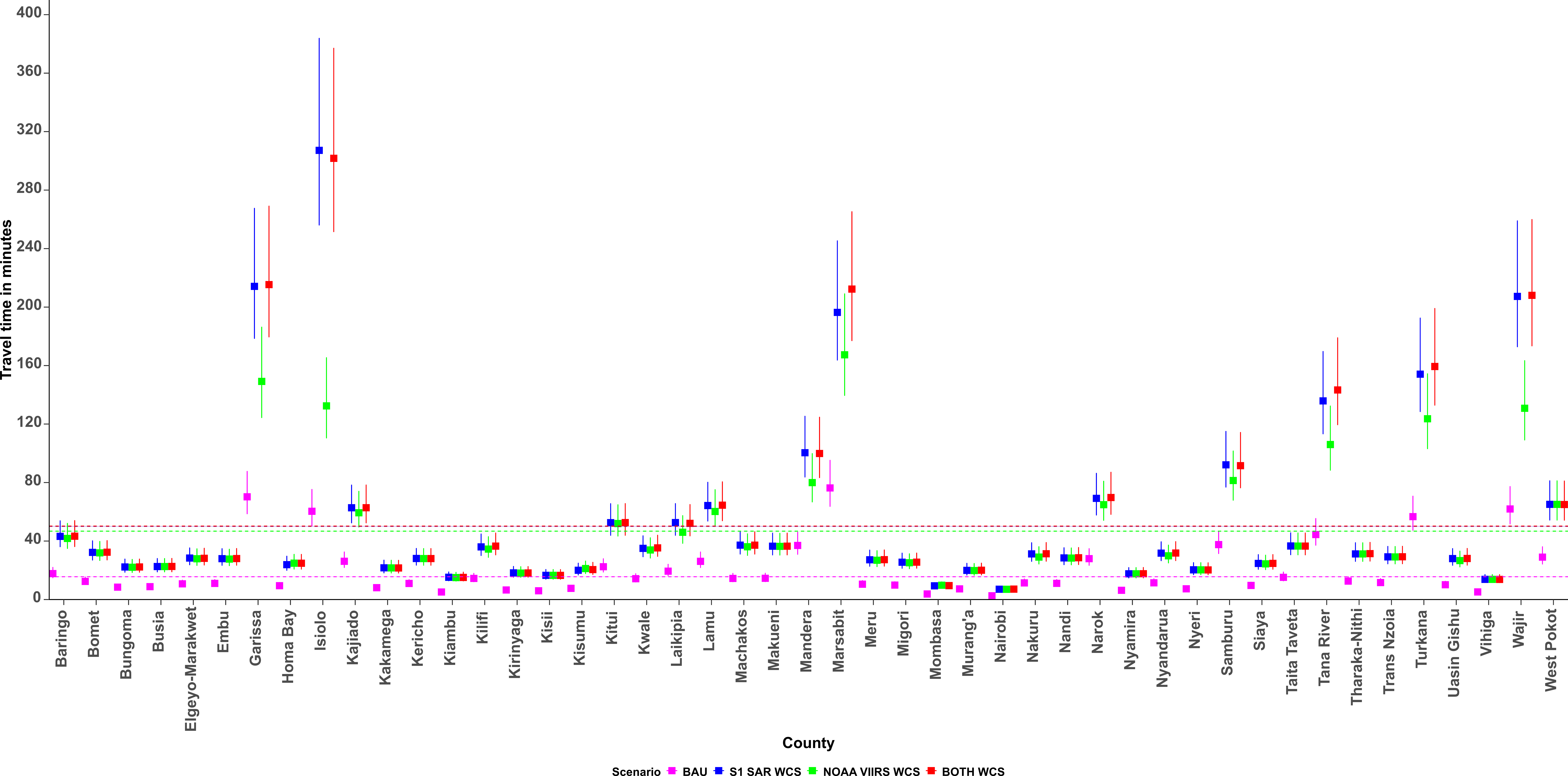


**Figure S6:** Boxplots at county level by scenario (lower average and upper bounds) for all facilities and average national travel time.

***Public Facilities***

Across public facilities (Figure S7), Nairobi had the shortest travel time: 3.8 minutes (3.3-4.7) for BAU, 11.1 minutes (9.3-13.7) for Sentinel 1 SAR, 11.0 minutes (9.3-13.6) for NOAA-VIIRS, and 11.2 minutes (9.4-13.9) for the worst-case. Marsabit had the highest average travel time for BAU 77.1 minutes (64.3-96.2) and NOAA-VIIRS 169.0 minutes (140.9-211.1), while Isiolo had the highest for Sentinel-2 310.3 minutes (258.7-387.8) and worst-case 305.1 minutes (254.3-381.3). Similarly, 13 and 14 counties were above the national average travel time under BAU and NOAA-VIIRS, respectively, while 12 were above for Sentinel 1 SAR and the combined scenarios.


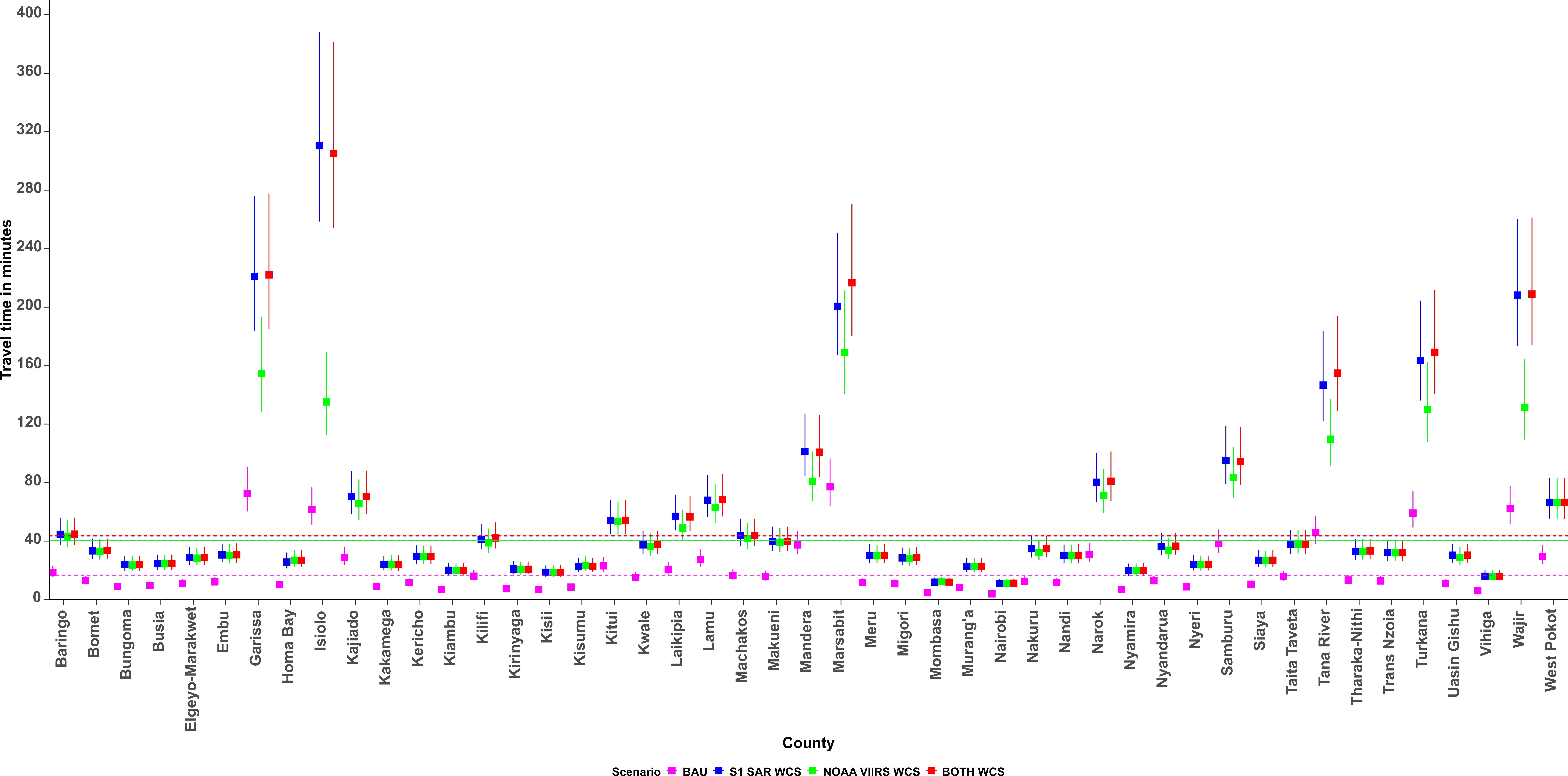


**Figure S7:** Boxplots at county level by scenario (lower average and upper bounds) for public facilities and average national travel time.

***Private Facilities***

Across private facilities (Figure S8), Nairobi recorded the shortest travel time to the nearest private facility across all scenarios: 2.9 minutes (2.5-3.5) for the BAU scenario, 7.9 minutes (6.7-9.8 ) for the Sentinel 1 SAR scenario, 7.8 minutes (6.6-9.7) for the NOAA-VIIRS scenario, and 8.1 minutes (6.8-10.0) for the combined scenarios. In contrast, Marsabit experienced the longest average travel time for each scenario, with 76.3 minutes (63.7-95.3) for the BAU scenario, 692.6 minutes (577.2-865.6) for the Sentinel 1 SAR scenario, 459.8 minutes (383.2-574.6) for the NOAA-VIIRS scenario, and 732.9 minutes (610.8-916.0) for the combined scenario. Thirteen counties had average travel times exceeding the national average to any facility in the BAU and NOAA-VIIRS scenarios, while nine counties surpassed the national average in the Sentinel 1 SAR and combined scenarios.


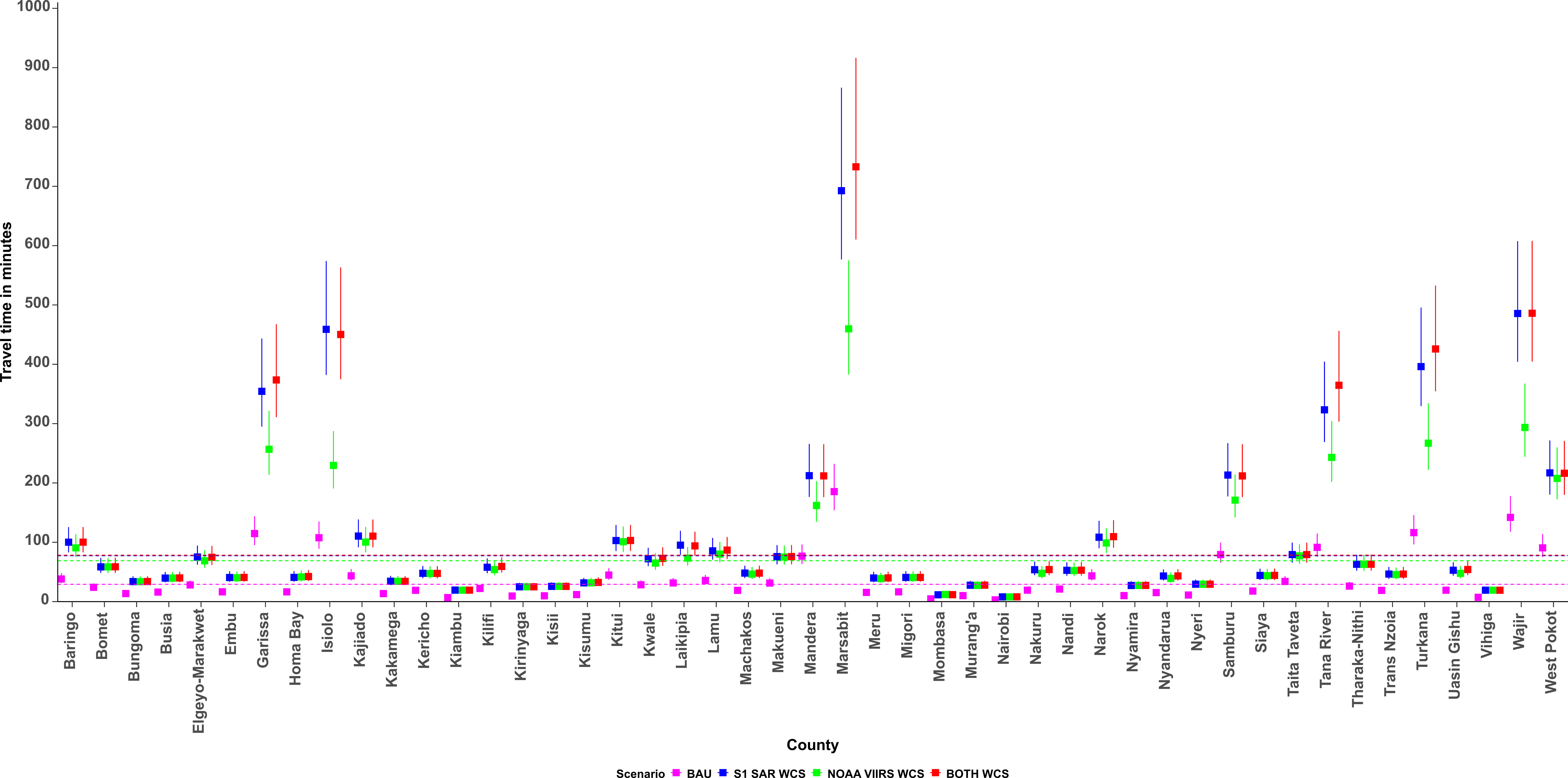


**Figure S8:** Boxplots at county level by scenario (lower average and upper bounds) for private facilities and average national travel time.

***Private-not-for-profit (PNfP) Facilities***

Nairobi had the lowest travel times to PNfP facilities (Figure S9): 4.1 minutes (3.5–4.9) for BAU, 10.9 minutes (9.2–13.5) for Sentinel 1 SAR, 10.9 minutes (9.2–13.5) for NOAA-VIIRS, and 10.9 minutes (9.1–13.4) for combined scenarios. In contrast, Mandera had the highest average travel times: 363.0 minutes (302.6-453.7) for BAU, 1,126.9 minutes (939.2-1,408.6) for Sentinel 1 SAR, 744.4 minutes (620.4-930.4) for NOAA-VIIRS, and 1,124.8 minutes (937.5-1,405.9) for combined. Twelve counties exceeded the national average travel time under BAU and NOAA-VIIRS, while eight counties surpassed the national average for Sentinel 1 SAR and combined scenarios.


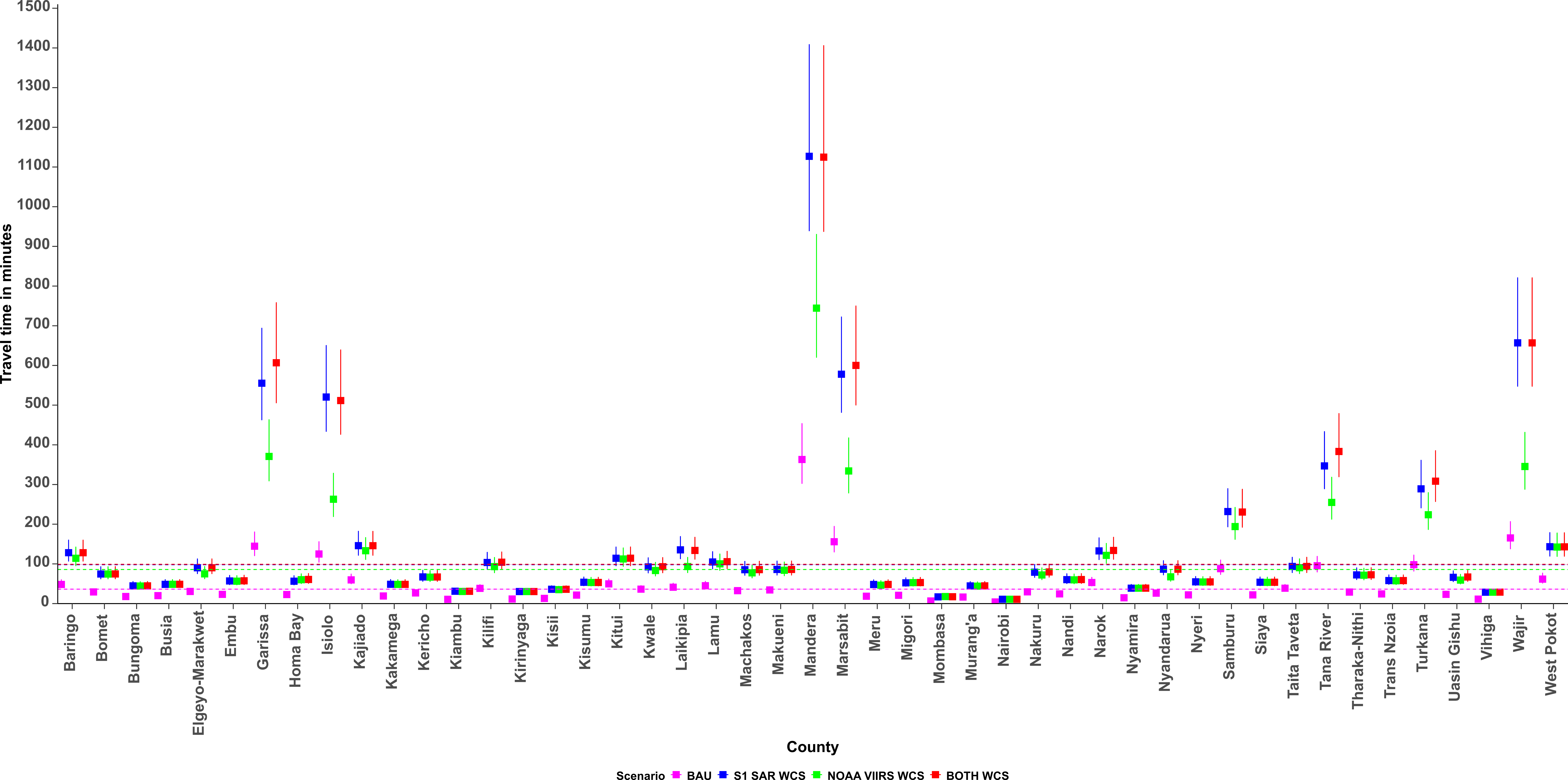


**Figure S9:** Boxplots at county level by scenario (lower average and upper bounds) for faith-based organisation facilities and average national travel time.

# **Population coverage pre- and post-floods per 30-min travel time band, across facility types and scenarios.**

***Public health facilities***

The proportion of population covered per travel time band and scenario for public health facilities is shown in Figure S10.


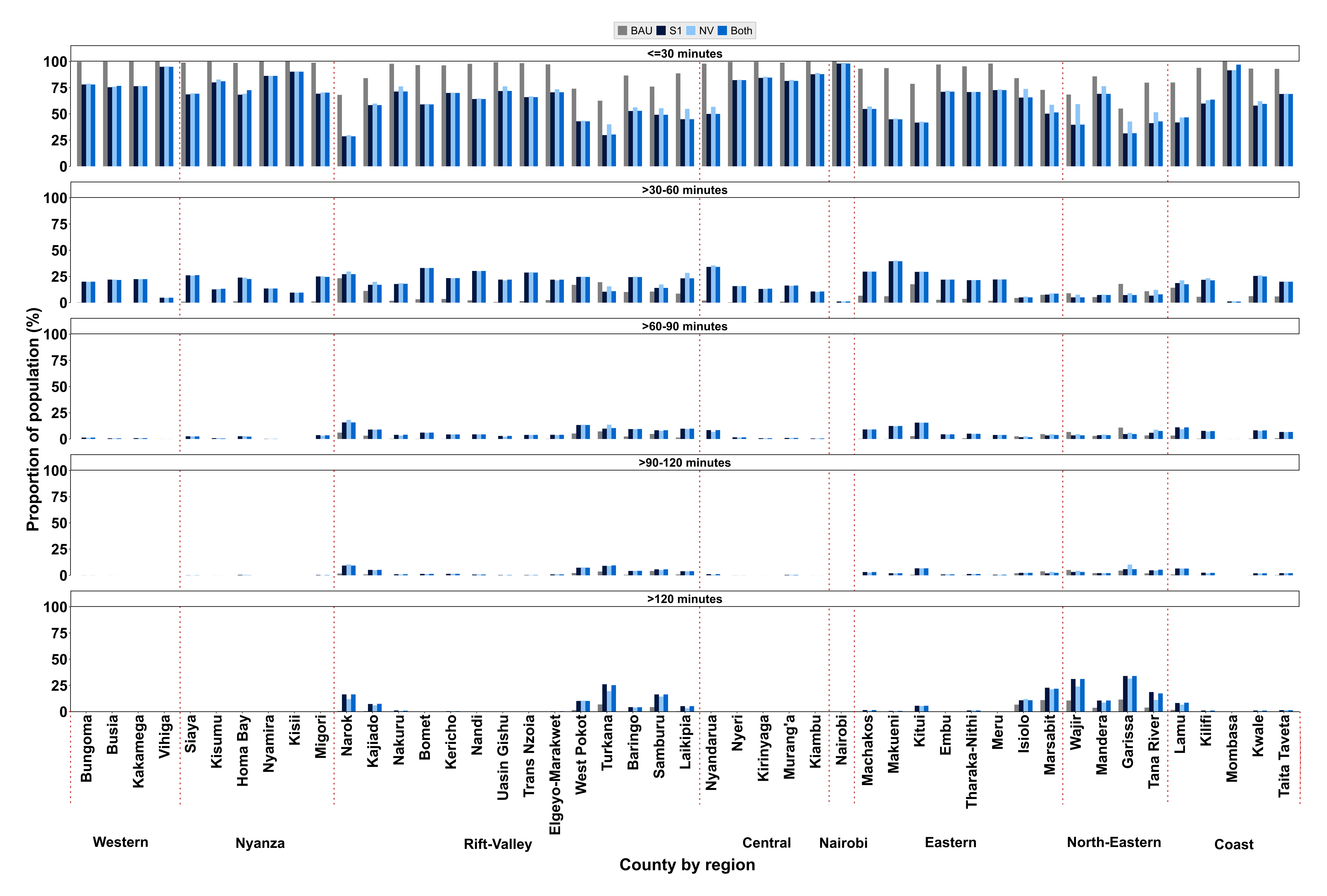


**Figure S10:** Percent population per travel time band and scenario for public health facilities

***Private health facilities.***

Figure S11 shows the proportion of population covered per travel time band and scenario for private health facilities across counties.





**Figure S11:** Percent population per travel time band and scenario for private health facilities.

***Private-not-for-profit (PNfP) Facilities.***

Figure S12 shows the proportion of population covered per travel time band and scenario for private health facilities across counties. Counties in arid and semi-arid regions that have the lowest numbers of PNfPs are the most affected.





**Figure S12:** Percent population per travel time band and scenario for PNfP health facilities.

# **Average travel time map at sub-county level by flood scenario and facility type.**

There was heterogeneity in access to health care pre and post floods across all sub-counties (Figure S13). For all and public health facilities under the BAU scenario, the average travel time was within 2 hours. For private and PNfP facilities, the average travel time for 16/298 and 23/298 sub-counties (majority in northeastern Kenya) were beyond 2hrs. For S2 flood scenarios, the average travel time for 25, 25, 48 and 59 of 298 sub-counties was above 2 hrs for all, public, private and PNfP facilities respectively. Similarly, for NOAA-VIIRS flood scenario, the average travel time for 18, 18, 44 and 52 of 298 sub-counties was above 2hrs for all, public, private and PNfP facilities respectively. Finally for the combined flooding scenario, the mean travel time for 25, 25, 48 and 59 of 298 sub-counties was above 120 minutes for all, public, private and PNfP facilities respectively.


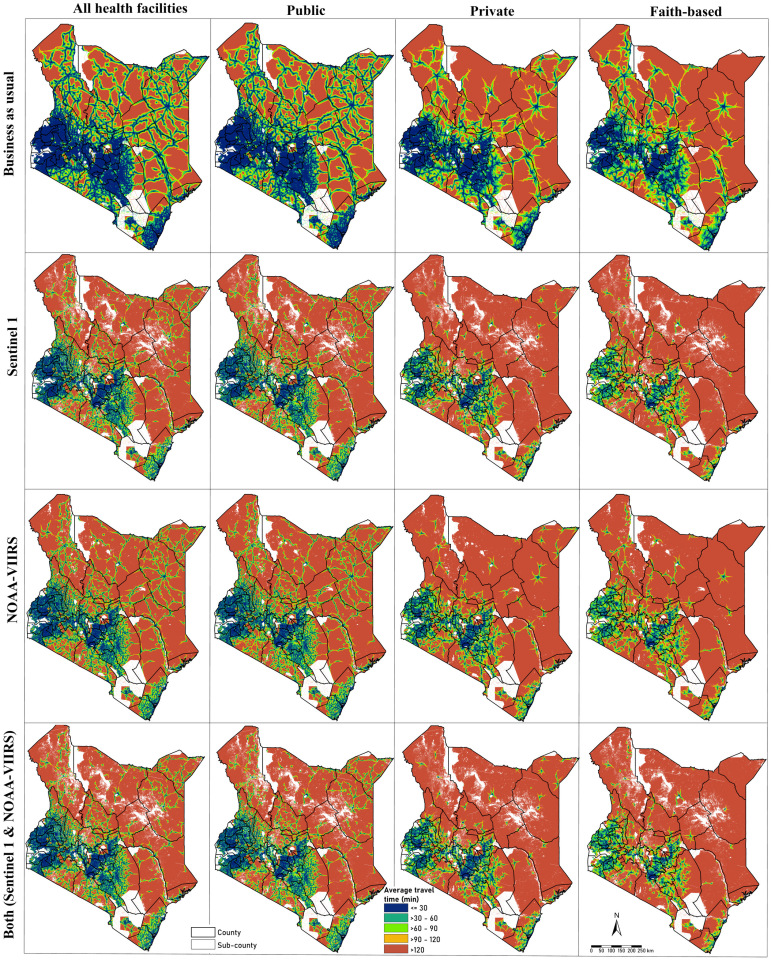


**Figure S13:** Classified/binned average travel time to the nearest health facility by facility type (all, public, private-not-for-profit, and private-for-profit) for the BAU and worst-case scenarios (Sentinel 1 SAR, NOAA-VIIRS, and the combined flooded extents.

**References**

1. KRB. KENYA ROADS BOARD (KRB) MAP PORTAL [Internet]. [cited 2024 Mar 1]. Available from: https://maps.krb.go.ke/kenya-roads-board12769/maps

2. Moturi AK, Suiyanka L, Mumo E, Snow RW, Okiro EA, Macharia PM. Geographic accessibility to public and private health facilities in Kenya in 2021: An updated geocoded inventory and spatial analysis. Front Public Health. 2022;10:1002975.

3. Macharia PM, Mumo E, Okiro EA. Modelling geographical accessibility to urban centres in Kenya in 2019. PLoS One. 2021;16(5):e0251624.

4. Karra K, Kontgis C, Statman-Weil Z, Mazzariello JC, Mathis M, Brumby SP. Global land use / land cover with Sentinel 2 and deep learning. In: 2021 IEEE International Geoscience and Remote Sensing Symposium IGARSS [Internet]. 2021 [cited 2025 Aug 23]. p. 4704–7. Available from: https://ieeexplore.ieee.org/document/9553499

5. RCMRD. Kenya SRTM DEM 30meters [Internet]. [cited 2024 Mar 1]. Available from: https://gmesgeoportal.rcmrd.org/datasets/rcmrd::kenya-srtm-dem-30meters/about

6. UNEP-World Conservation Monitoring Centre (WCMC), International Union for Conservation of Nature (IUCN). The world database on protected areas. 2023 [cited 2023 Dec 9]. About. Available from: https://www.protectedplanet.net/en/about

7. WorldPop. WorldPop, open data for spatial demography | Scientific Data [Internet]. [cited 2025 Aug 23]. Available from: https://www.nature.com/articles/sdata20174
